# Substantially lower estimates in China’s offshore wind potential using farm-scale spatial modeling and wake effects

**DOI:** 10.1038/s41467-026-68655-2

**Published:** 2026-01-26

**Authors:** Shiwei Xu, Gege Yin, Peiyu Hu, Di Dong, Yue Qin, Yu Liu, Gang Liu, Lili Song, Chuan Zhang

**Affiliations:** 1 Institute of Energy, Peking University, Beijing, China; 2 School of Earth and Space Sciences, Peking University, Beijing, China; 3 College of Urban and Environmental Sciences, Peking University, Beijing, China; 4 College of Environmental Science and Engineering, Peking University, Beijing, China; 5 Chinese Academy of Meteorological Sciences, Beijing, China; 6 Institute of Carbon Neutrality, Peking University, Beijing, China

**Keywords:** Wind energy, Climate change, Energy infrastructure

## Abstract

Renewable energy is critical for addressing global climate change, and accurate assessments of its potential are key for decision making and planning. This study provides a detailed, farm-level evaluation of offshore wind power potential in China, incorporating realistic turbine layouts derived from remote sensing data, wake loss modeling, and future climate scenarios. Our findings show that accounting for the farm-level details results in a China’s offshore wind potential of 2.5–4.2 PWh yr^−1^ which is significantly lower than previous estimates, which often exceeded 5.6 PWh yr^−1^. Through modeling the wake loss effects within wind farms, the study reveals that wake losses are higher than previously assumed in earlier research. Additionally, the study highlights substantial economic and technical disparities between nearshore bottom-fixed and deep-water floating wind farms, with the latter offering higher potential density but at greater costs. Our results provide a more realistic foundation for setting energy targets, optimizing regional strategies, and promoting floating wind technologies to harness deep-water resources, thereby supporting China’s transition to a sustainable energy future.

## Abstract (web summary)

The study evaluates China’s offshore wind potential using farm-scale modeling, revealing lower potential than previous estimates. It incorporates realistic turbine layouts, wake loss modeling, and climate scenarios, highlighting economic and technical differences between nearshore and deep-water wind farms.

## Introduction

The continuous growth of global energy demand, coupled with increasing climate change issues, highlights the critical importance of utilizing renewable energy sources^1^. Among various renewable energies, offshore wind power has been widely acknowledged as an indispensable element in future net-zero energy systems. International Renewable Energy Agency (IRENA) projects that 2465 GW of offshore wind would be needed in 2050 worldwide, with 8000 TWh power generation^2^, which accounts for 10% of global power demand. In China, offshore wind power has witnessed a huge leap during the past decades as well, namely 34.4 GW capacities have been added from 2010 to 2020^3^, contributing 5% of the national emission-free power generation for that period. However, the development of offshore wind in China is facing new challenges: resource exploitation from nearshore wind has been almost finished, and the push of offshore wind to deeper water and longer offshore distances is becoming the new trend^4^. In light of such industry and policy advancements, the assessment of offshore wind resources also needs new calibrations.

Resource assessment of wind energy typically adopts a geographically bottom-up siting method, which firstly identifies a list of suitable sea areas for potential wind power exploitation considering exclusion criteria like water depth, wave, and protected area^5^, then uniformly spaces selected wind turbines across all available areas to calculate the theoretical power generation potential across such areas with naïve wake loss factors (Fig. S1 illustrates the classic spacing rules for wind turbines)^6^. Several shortcomings come with such an assessment framework, for example, existing assessments primarily start with the scale of individual turbines, whereas actual offshore wind farms are usually planned on a larger, farm-scale, with their sizes continually expanding^3,7^. Also, current evaluation methods lack sufficient practical references for turbine spacing (typically assumes *7D* * *7D*, where *D* is the rotor diameter of the selected wind turbine)^8,9^ and deploy a rough representation of wake effects within wind farms (typically assumes 10%)^10,11^, leading to inaccuracies in potential assessment. The use of wind speed data in these studies is not consistent as well, recently a couple of works have quantified the impact of climate change on global wind power under different scenarios, yet such results are still coarse for power system planning^12,13^. All the above issues constitute the rationale for conducting a new offshore wind power potential assessment in our study.

**Fig. 1:**
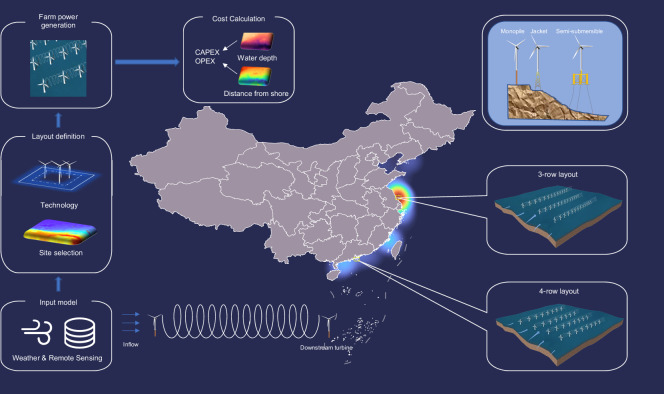
Farm-level offshore wind power potential in China considering optimal turbine layout, wake effect, and future wind speeds. **S**hown in the middle of the figure is the distribution of existing offshore wind farms in China, whereas the left shows the flowchart of our method, and the right shows different installation methods for wind turbines and the related wind farm layout in the paper.

To achieve a more accurate assessment of offshore wind resources, it is necessary to consider real-world design requirements and wake effects from a farm-level perspective while accounting for future wind speed changes under different climate change scenarios^14,15^. This study aims to provide a realistic and spatially explicit assessment of China’s offshore wind power potential under both current and future climate conditions. To achieve this, we integrate wind farm-scale turbine layout statistics derived from remote sensing data, multiple wake loss models, and a bias-corrected CMIP6 climate dataset. As shown in Fig. 1, we first assessed the windward and vertical spacing of turbines in existing offshore wind farms in China to obtain realistic spacing standards and identified the realistic layout patterns through remote sensing data. On this basis, we set up wind farm design patterns with different farm scales and wind turbine types, then filter wind farm construction based on various weather, ocean, and geographical data, and place best practice wind farms in all available areas. Subsequently, we calculated the annual power generation of these wind farms under both historical and future wind speed datasets, with dedicated calculation of farm-level wake effect losses. This comprehensive approach provides a farm-level assessment of the potential for offshore wind power in China. In addition, we formulated a levelized cost of electricity (LCOE) model based on the specific location of turbines in each wind farm (reflecting water depth and offshore distance) to estimate the economic feasibility of these wind farms, thus determining construction priorities. An overview of the entire process can also be found in Fig. S2.

In the work, the results indicate that if assessing the resource potential of offshore wind power in China based on realistic farm design, turbine spacing, and wake loss, the resulting potentials are much lower than previously predicted in all studies. The LCOE of offshore wind farms, particularly floating offshore wind farms, also becomes much higher than previously projected, necessitating a re-evaluation of offshore wind’s techno-economic competitiveness in China. On the other hand, this work introduces a new framework for wind farm-scale offshore resource assessment, offering scientific insights into the spatial, technical, and economic dynamics of wind energy deployment.

## Results

### Assessment of wind farm layout and turbine spacing

The assessment of offshore wind resources in China primarily focuses on selecting appropriate wind datasets, considering physical constraints, estimating power generation costs, and predicting future power output and costs^16–19^. While these assessments play an important role in policy-making and strategic planning, the design of turbine spacing is often estimated in a simplified manner, and many assessments are conducted at the individual turbine level^20–23^. This narrow focus frequently misaligns with the practical realities of offshore wind farm construction and operation. To address this gap, we conducted an evaluation of turbine spacing and farm-scale configurations for offshore wind farms in China.

**Fig. 2:**
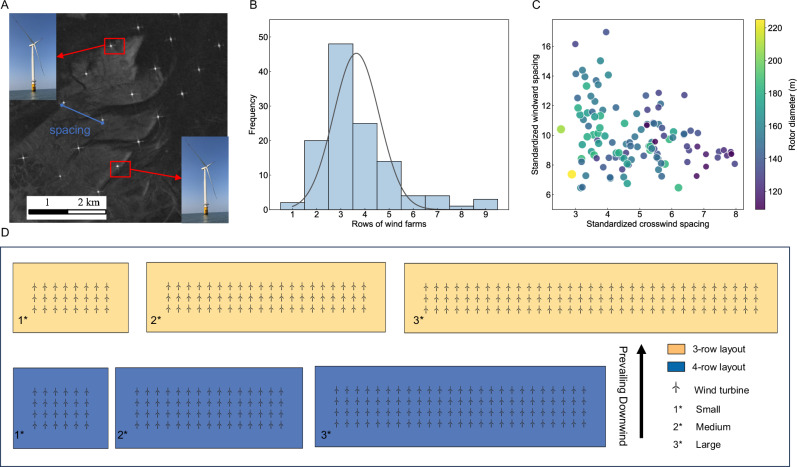
Current wind-farm layout and turbine spacing in China. Shown in this figure are: **A** examples of remote sensing images of offshore wind farms in China^64^, **B** distribution of existing wind farm row numbers (denoted by prevailing wind direction) in China, **C** standardized spacing of wind farms in current wind farms among prevailing direction, **D** typical wind-farm layouts identified through remote sensing analysis of existing wind farms in China.

We conducted a comprehensive assessment of the wind turbine layout and spacing for the majority of existing wind farms in China as of 2022 (remote sensing images of two of these farms are shown in Fig. 2A). The distribution of the rows and the fitted Gaussian curves in Fig. 2B show that the majority of wind farms in China mainly adopt the three-row layout, followed by the four-row layout. These two arrangements represent 39.7% and 20% of the wind farms included in the study, respectively. And Fig. 2C depicts the distribution of windward spacing and crosswind spacing for each wind farm, normalized by the rotor diameter of the turbines within the farms. The data reveal that windward spacing predominantly ranges between 8 and 12 rotor diameters, while the crosswind spacing spans from 3 to 6 rotor diameters (see Table S1 for detailed data). Based on these parameters and integrating the wind farm sizes from IRENA^24^, we designed six wind farm configurations as illustrated in Fig. 2D, considering both 3-row and 4-row layouts for small (24 turbines), medium (60 turbines), and large farms (99 turbines for 3-row and 100 turbines for 4-row layouts). For further details, refer to the Materials and Methods and the Supporting Information.

### Technical potential of offshore wind power in China

**Fig. 3:**
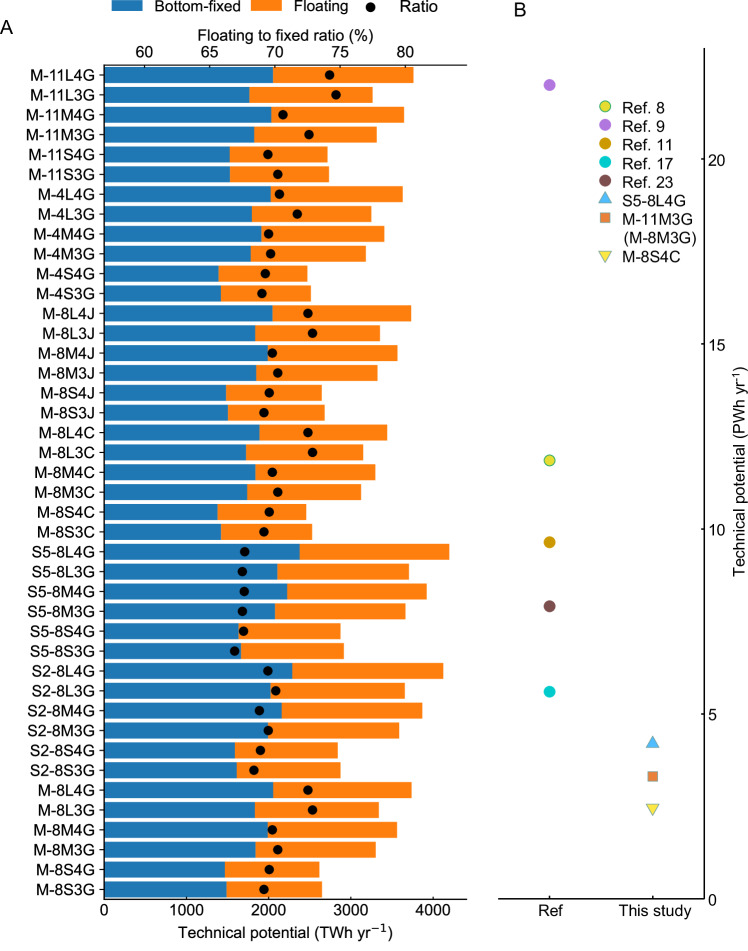
Estimated China’s offshore wind power potential across different scenarios. Subfigure (**A**) displays the category of scenarios approached in this work, considering various wind speed data, turbine ratings, wind farm sizes, layouts, wake models, together with corresponding power generation potential, covering both bottom-fixed and floating installations, detailed parameter settings are provided in Table S4. Whereas subfigure (**B**) is the comparison of this study’s offshore wind power potential estimation with previous assessments^8,9,11,17,23^. The three points in subfigure (**B**) represent the maximum (S5-8L4G, a 4196TWh/year potential calculated by SSP5-8.5 wind speed data, 8 MW turbine size, 4-row large-scale farm, and Gaussian wake effect method), median (M-11M3G & M-8M3G), and minimum (M-8S4C) values across all scenarios in this study.

In this study, we establish realistic spacing criteria by analyzing turbine spacing in existing offshore wind farms. We then expand the scope of our assessment from individual turbines to entire wind farms. By integrating factors such as wind farm size, turbine capacity, layout pattern, and spacing standards, we use NASA’s MERRA-2 (Modern-Era Retrospective Analysis for Research and Applications, Version 2) reanalysis dataset, along with a bias-corrected dataset from the Coupled Model Intercomparison Project Phase 6 (CMIP6) for dynamic downscaling of both historical and future climate scenarios (SSP2-4.5 and SSP5-8.5)^25,26^. We selected three types of wind turbines with different rated powers based on data from existing wind farms and used three different wake calculation models to assess wake losses under each scenario (see Materials and Methods for details). Figure 3A illustrates the total offshore wind power potential of China under various scenarios, the potential ranges from 2.62 to 3.74 PWh yr^−1^, 2.84 to 4.12 PWh yr^−1^, and 2.87 to 4.20 PWh yr^−1^ across three wind speed datasets, indicating relatively high technical potential in future climate scenarios. Under the same wind speed data (MERRA-2), different wake models also result in variations in potential. Compared to the Gaussian velocity model, the potential using the classic Jensen model shows little change, while the potential with the cumulative curl model is relatively lower. The potential ranges for these two models are 2.64 to 3.73 PWh yr^−1^ and 2.46 to 3.44 PWh yr^−1^, respectively. Additionally, when the wind speed data and wake model remain consistent, changes in turbine rated power also impact national power generation potential. When the rated power is 4 MW, the power generation potential ranges from 2.47 to 3.63 PWh yr^−1^, whereas with a rated power of 11 MW, the range is 2.72 to 3.76 PWh yr^−1^.

From the perspective of turbine installation type, the total generation potential of fixed installations is generally higher than that of floating installations in most scenarios. However, as shown in Fig. 3A, the number of floating wind farms is only 65% to 75% of the number of bottom-fixed wind farms, resulting in a much higher overall potential density for floating wind turbines compared to bottom-fixed wind farms. By comparing our results with previous studies (Fig. 3B), we found that even under the less restrictive criteria we consider (Table S2), the results are still substantially smaller than those suggested by previous studies (Table S3). Specifically, the median potential has been reduced from roughly 10 PWh yr^−1^ in previous studies to below 5 PWh yr^−1^ in this analysis.

From a provincial perspective, the offshore wind technical potential of China’s coastal provinces varies greatly. Table 1 shows the wind power potential in each province under three typical scenarios, provinces such as Zhejiang, Guangdong, Hainan, Jiangsu, and Shandong exhibit large power generation potential under various scenarios, with median values above 350 TWh yr^−1^. In contrast, provinces such as Guangxi, Hebei, and Shanghai have relatively low offshore wind power potential due to limited sea area and poor wind speed conditions, with median values below 80 TWh yr^−1^. The distribution of provincial generation potential is more consistent across scenarios, but the SSP2-4.5 dataset and SSP5-8.5 dataset will have slightly higher generation potential than the MERRA-2 dataset. Moreover, except for Hainan and Zhejiang provinces, the offshore wind power potential in these regions is generally insufficient to meet their respective electricity demands. Most provinces can only generate 60-80% of their power requirements, with Shanghai and Hebei having even lower generation potential, meeting less than 20% of their electricity needs. This disparity underscores the need for a strategic reassessment of offshore wind resource development and utilization to better optimize their contribution to regional energy needs.

**Table 1:** Comparison of power consumption and offshore wind power potential in different provinces

Province	Electricity consumption (TWh)	Offshore wind farm potential (TWh)		
		M-8M3G	S2-8M3G	S5-8M3G
Fujian	290	173	181	181
Guangdong	787	481	584	580
Guangxi	222	45	47	50
Hainan	42	706	790	791
Hebei	434	56	65	69
Jiangsu	740	371	396	456
Liaoning	255	204	197	205
Shandong	756	436	435	436
Shanghai	175	26	33	32
Zhejiang	580	802	859	863
Total	4280	3299	3587	3662

Demand data by province are from the National Bureau of Statistics^79^.

### Farm-level cost of offshore wind

**Fig. 4:**
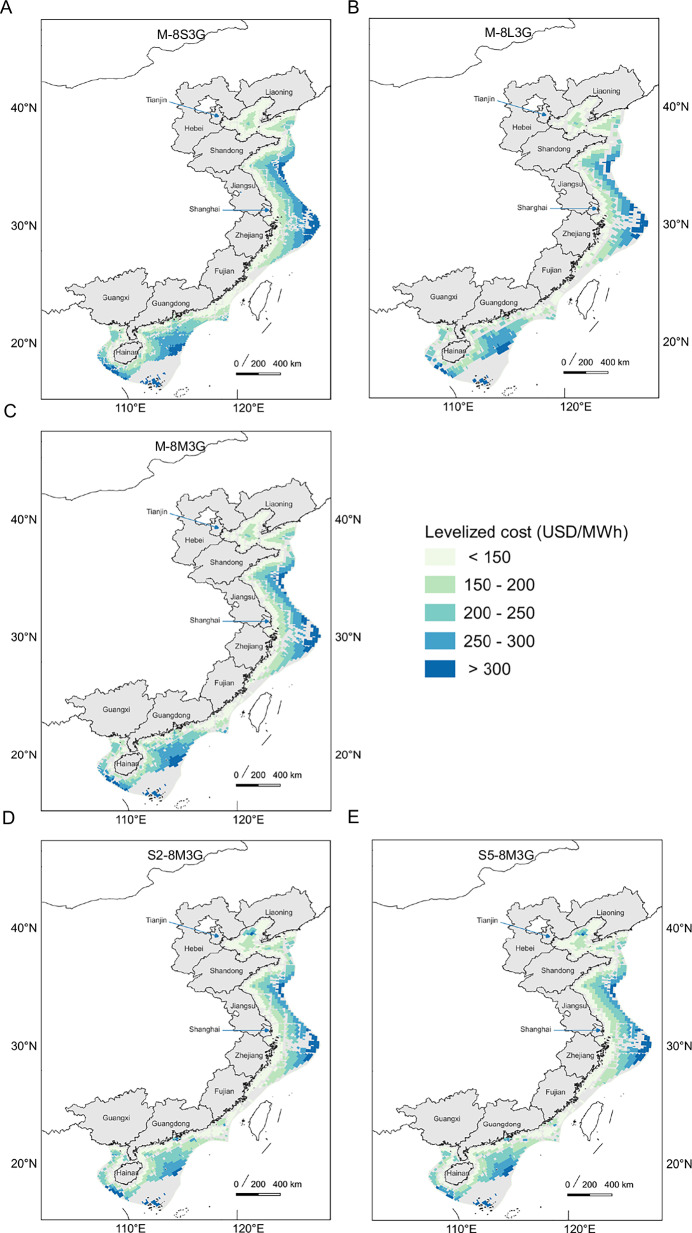
Spatial distribution of LCOE for offshore wind farms in China. Compared in this figure are the spatial distribution of farm-level offshore wind power LCOE in China (based on 3-row layout), with subfigures (**A**–**C**) representing the M-8S3G, M-8M3G, M-8L3G scenarios, highlighting the impact of wind farm scale on LCOE, **C**–**E** representing the M-8M3G, S2-8M3G, S5-8M3G scenarios, focusing on the influence of different wind speed datasets on LCOE. Scenarios for the 4-row layout are presented in Fig. S7.

To further assess the cost and distribution patterns of offshore wind power, we conducted an economic evaluation of wind farms that meet construction standards, incorporating existing cost data, water depth, and distance from shore (details in Materials and Methods and Supporting Information). Figure 4A–C presents the wind farm distribution and economic evaluation results for three scenarios with identical settings, differing only in wind farm scale (M-8S3G, M-8M3G, M-8L3G). Similarly, Fig. 4C–E displays the wind farm distribution and economic evaluation results for three scenarios with identical settings, except for the wind speed data (S2-8M3G, M-8M3G, S3-8M3G), under varying wind speed conditions. The figures show that while wind speeds generally improve with greater distance from the coast, water depth also increases, leading to a significant rise in installation, transmission, and operation and maintenance costs. Consequently, the LCOE for wind farms in the scenarios presented tends to increase proportionally with distance from shore. Low-cost areas are primarily concentrated along the coasts of Jiangsu and Zhejiang, whereas provinces like Guangdong and Hainan show a higher proportion of high LCOE. By comparing the LCOE distribution map with the capacity factor map (Fig. S6 for the 3-row layout, Fig. S8 for the 4-row layout), it becomes clear that higher capacity factors do not always correspond to lower LCOE, due to the combined effects of water depth and distance from shore. For instance, the coastal areas of Fujian and Zhejiang exhibit lower LCOE with higher capacity factors, a relationship that is not observed in the coastal areas of Guangdong and Shandong.

**Fig. 5:**
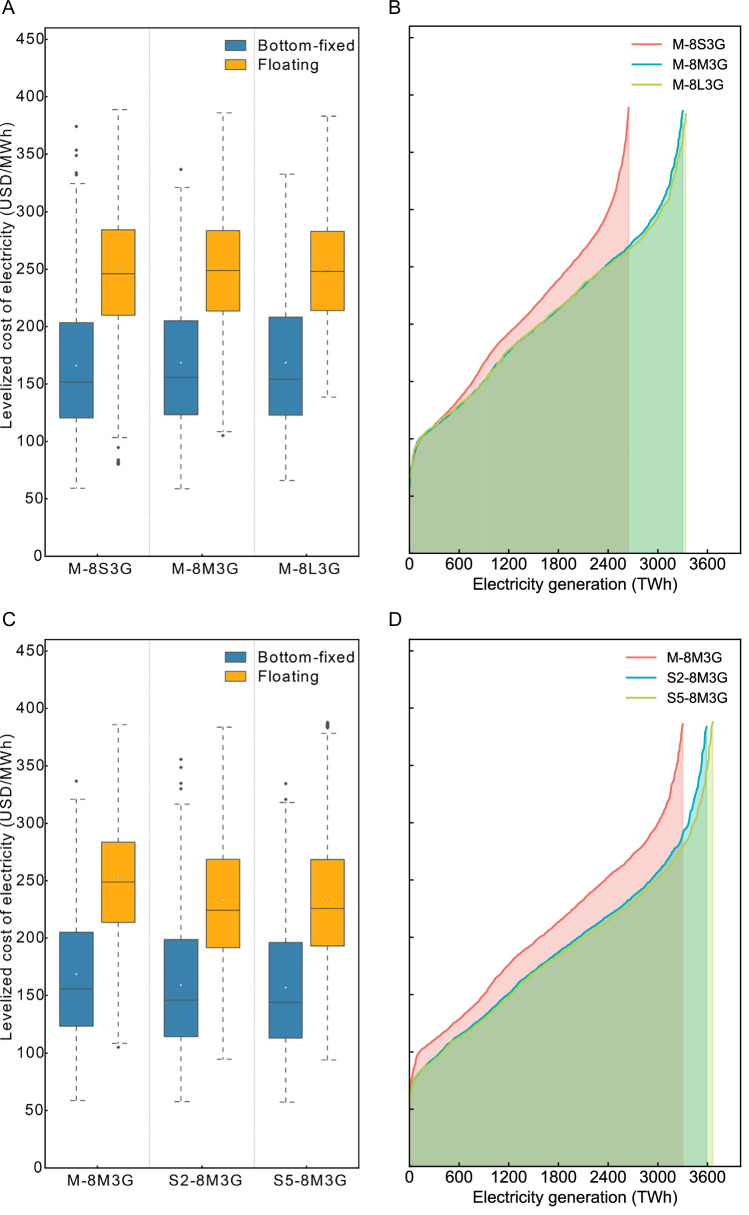
LCOE and aggregated offshore wind power supply curves in China. This figure illustrates the comparison of LCOE and aggregated offshore wind power supply curves for different scenarios in China, based on a 3-row wind farm layout. Subfigures (**A**, **B**) assess the impact of wind farm scale on LCOE and corresponding supply potential, covering scenarios M-8S3G, M-8M3G, and M-8L3G. Subfigures (**C**, **D**) examine the influence of different wind speed datasets (MERRA-2, SSP2-4.5, and SSP5-8.5) on LCOE and electricity supply under consistent turbine configuration and farm size. The boxplots show the distribution of LCOE values for bottom-fixed and floating installations, while the supply curves indicate the cumulative electricity generation achievable below a given cost threshold. Scenarios with 4-row layouts are provided in Fig. S7.

From the perspective of changes in the size of wind farms (Fig. 4A–C), it is evident that small-scale wind farms are more numerous and densely concentrated. However, as the size of the wind farms increases, both the number and density of wind farms decrease significantly. Figure 5A, B illustrates the cost distribution for bottom-fixed wind farms, floating wind farms across the three scenarios bottom-fixed wind farms exhibit significantly lower costs than floating wind farms, with the majority of bottom-fixed wind farm costs falling below 125 USD/MWh. In contrast, floating wind farms have costs primarily in the range of 130 to 160 USD/MWh. The overall cost distribution for both types of wind farms lies between 100 and 160 USD/MWh, indicating that relatively few wind farms are currently economically viable. Figure 5A, B also shows that as the wind farm scale expands, the corresponding LCOE decreases accordingly. Furthermore, it clearly demonstrates that as total power generation increases, the LCOE for small-scale wind farms (M-8S3G) rises more rapidly and becomes much higher than that of medium-scale and large-scale wind farms (M-8M3G, M-8L3G). Notably, LCOE for small-scale wind farms is only comparable to that of medium-scale and large-scale wind farms during the initial small fraction of generation.

From the perspective of different wind speed data, the distribution of wind farms and their corresponding LCOE values remains relatively consistent across the scenarios presented. Notably, under the future climate scenarios (S2-8M3G, S5-8M3G), overall wind farm costs are lower, whereas the MERRA-2 scenario (M-8M3G) results in higher costs. It can also be seen in Fig. 5C, D that the M-8M3G scenario results in higher cost estimates compared to the S2-8M3G and S5-8M3G scenarios, with the median cost being approximately 10 USD/MWh higher than the other scenarios for both bottom-fixed and floating wind farms. In addition, the national cumulative supply curves for these three scenarios show that, in the M-8M3G scenario, the cost for the first 200 TWh of cumulative generation potential is relatively low, around 80 USD/MWh, but then rises rapidly for subsequent capacities, with a sharp increase after 3000 TWh. In contrast, for the S2-8M3G and S5-8M3G scenarios, costs gradually rise to 75 USD/MWh for the first 600 TWh of cumulative generation, then increase rapidly from 600 TWh to 1000 TWh, followed by slower growth until 3000 TWh, after which costs rise sharply.

### Wake loss effect

Previous studies have often chosen to either ignore wake losses or apply a generalized reduction factor using a fixed coefficient. Recently, more advanced modeling efforts have been developed to resolve wake interactions at the turbine level, particularly for site selection purposes. Gil-García et al.^27,28^, for example, implemented GIS-based MCDM approaches to optimize turbine placement while accounting for wake-induced efficiency losses. Pryor et al.^29^ also employed mesoscale simulations to quantify the regional wake shadow impacts of large offshore wind clusters in the U.S., demonstrating how large-scale wake effects can influence wind availability at the lease-area scale. However, they do not quantify the techno-economic implications of wake losses across large-scale offshore wind deployments. In contrast, this study calculates wake losses in wind farm power generation using the Gaussian velocity model^30,31^ in FLORIS^32^, which describes the velocity deficit as a Gaussian distribution in the spanwise direction. Additionally, we supplemented this approach with both the Jensen model^33^ and the cumulative curl model^34^ for a more comprehensive analysis.

**Fig. 6:**
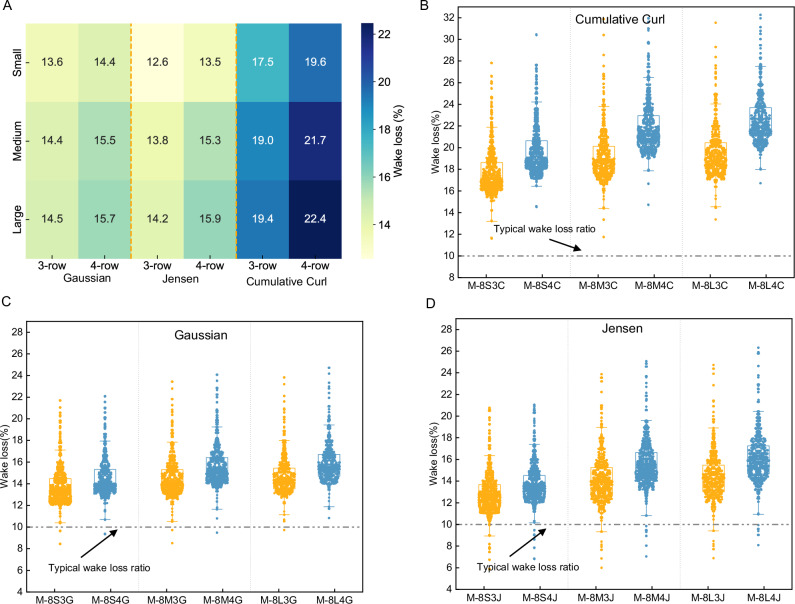
Impact of wake effects on the power generation potential of offshore wind farms. **A** shows the overall wake losses under different wake models and different wind farm scenarios, **B**–**D** display the distribution of wake losses for wind farms calculated using the Cumulative Curl model, Gaussian model, and Jensen model, respectively. The typical wake loss rate lines referenced from previous literature^10,11,35^.

Figure 6A shows the overall wake losses under different wake models and wind farm scenarios. It is evident that, within wind farms of the same size, the 4-row layout typically experiences higher wake losses than the 3-row layout. This is because increasing the number of turbine rows intensifies the wake effect, causing more downwind turbines to be affected, thereby reducing overall efficiency. For instance, in medium-sized wind farms using the Gaussian model, the total wake loss for the 3-row layout is 14.4%, while it increases to 15.5% for the 4-row layout. Additionally, within the same layout, the larger the wind farm, the greater the total wake loss. For example, in wind farms using the Jensen model, wake losses increase progressively in the 3-row layout from 12.6% in small wind farms to 13.8% in medium wind farms and 14.2% in large wind farms. Similarly, in the 4-row layout, wake losses rise from 13.5% in small wind farms to 15.3% in medium wind farms and 15.9% in large wind farms.

On the other hand, when comparing the three wake models, the Gaussian and Jensen models exhibit similar wake losses, while the Cumulative Curl model shows significantly higher losses. For example, in the medium-sized wind farm scenario with a 3-row layout, wake loss increases from 14.4% in the M-8M3G scenario and 13.8% in the M-8M3J scenario to 19.0% in the M-8M3C scenario. The wake effect’s impact on overall resource assessment in all scenarios is greater than the previously commonly used overall decay coefficients^10,11,35^. In addition, floating wind farms have lower wake losses than fixed wind farms, which is mainly due to the fact that floating wind farms have better wind conditions, and therefore higher power production even after wake attenuation (Fig. S9).

Figure 6B–D illustrates the distribution of wake losses across different wind farm configurations, highlighting a clear concentration of these losses within each scenario. In the Cumulative Curl model, wake losses in the 3-row layout are primarily concentrated between 16.4% and 20.5%, while in the 4-row layout, losses range from 18.2% to 23.7%. In contrast, the wake loss distribution under the Gaussian and Jensen models shows less variation across scenarios. Specifically, in the 3-row layout, wake losses are generally distributed between 11.7% and 15.5%, while in the 4-row layout, the values range from 12.5% to 15.5%. The concentration of wake losses in wind farms calculated using the Gaussian and Jensen models is notably narrower compared to the Cumulative Curl model.

### Importance of farm-scale analysis

**Fig. 7:**
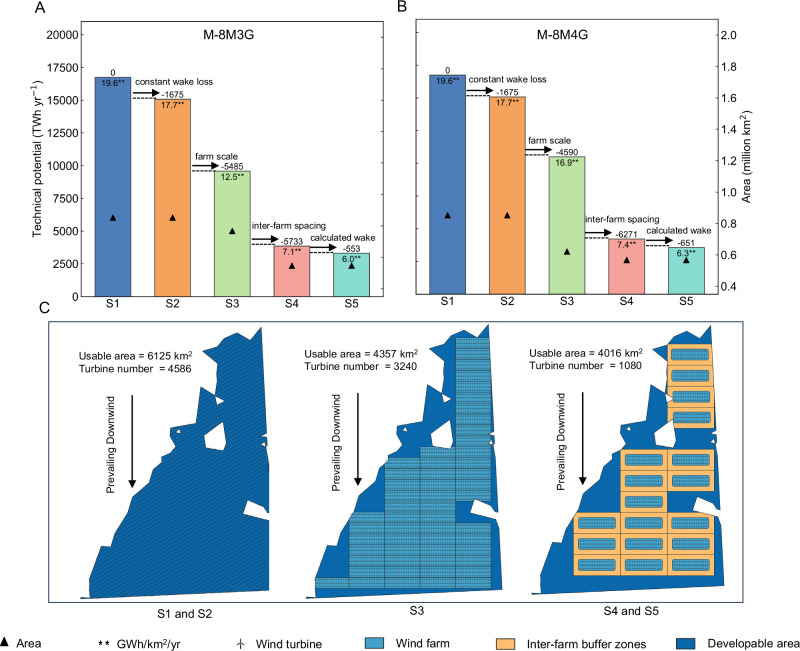
Changes in national offshore power generation due to different reasons. **A**, **B** Changes in national offshore power generation potential due to parameter adjustments in two typical scenarios(M-8M3G, M-8M4G): S1 is the result calculated using the unit area installed density method without considering wake losses; S2 is the result calculated using the unit area installed density method with estimated wake losses (10%); S3 is the result calculated at the farm scale without considering inter-farm buffer zones and estimating wake losses (10%); S4 is the result calculated at the farm scale considering inter-farm buffer zones but without wake losses; and S5 is the result calculated at the farm scale considering both inter-farm buffer zones and wake losses. **C** Schematic illustration of the calculation methodology applied in different scenarios in (**A**, **B**), using a representative region under the M-8M3G scenario as an example. Case scenarios of SSP2-4.5 and SSP5-8.5 are shown in Fig. S11.

In the transition from single-turbine assessment (MW/km^2^) to farm-scale offshore wind resource assessment, various factors play crucial roles, which are thoroughly analyzed in this study. We conducted a detailed analysis of the factors influencing the variation in generation potential for two typical scenarios (M-8M3G and M-8M4G). As shown in Fig. 7A, B, conditions S1 and S2 use methods from previous literature to calculate the offshore wind power potential per unit area density. S1, which does not consider wake effects, yields a potential value of 16.8 PWh yr^−1^, and S2, which accounts for estimated wake attenuation (10%), yields a potential value of 15.2 PWh yr^−1^; the values are the same in both scenarios. These values are within the range of previous literature estimates of potential^8,9,11,17,23^. We then introduced S3 and S4. Condition S3 was calculated at the farm scale without considering inter-farm buffer zones and estimating wake losses (10%), resulting in a potential value of 9.6 PWh yr^−1^ in M-8M3G and 10.5 PWh yr^−1^ in M-8M4G. S4 considers inter-farm buffers but not wake losses, resulting in a potential value of 3.9 PWh yr^−1^ in M-8M3G and 4.2 PWh yr^−1^ in M-8M4G. Finally, in S5, the potential drops further to 3.3 PWh yr^−1^ in M-8M3G and 3.6 PWh yr^−1^ in M-8M4G, approximately 16.7% and 15.5% lower than the S4 without considering wake losses. Comparatively, the M-8M4G scenarios exhibit a higher generation potential than the M-8M3G scenarios under S3, S4, and S5 conditions, typically reflected in their greater annual energy production per unit area. The changes in power generation potential for each province across all scenarios are presented in Fig. S10.

The available area for single-scale wind turbines in both scenarios, represented by the S1 and S2, is 0.85 million km². At the farm-level scale, represented by S3, the available area decreases to 0.76 million km² in M-8M3G scenarios and 0.62 million km² in M-8M4G scenarios. After increasing the spacing between wind farms, the available area remains relatively stable, with S4 and S5 conditions both having an available area of 0.55 million km² in M-8M3G scenarios and 0.57 million km² in M-8M4G scenarios. Using different calculation methods will lead to significant changes in the number of wind turbines that can be installed and the available area. These changes are shown schematically in Fig. 7C (a typical area in the M-8M3G scenario), using an approximately infinite area farm (single-turbine perspective). In this area, the number of wind turbines that can be installed is 4586, and the available area is 6125 km^2^. From a farm-level perspective, the number of wind turbines that can be installed without a buffer zone is 3240, and the available area is 4357 km^2^. The number of wind turbines that can be installed with a buffer zone is 1080, and the available area is 4016 km^2^. A precise assessment of the area occupied by a wind farm is crucial^36^, highlighting the importance of farm-level scale planning due to the differences in the area mentioned above.

### Potential variations across different scenarios analysis

**Fig. 8:**
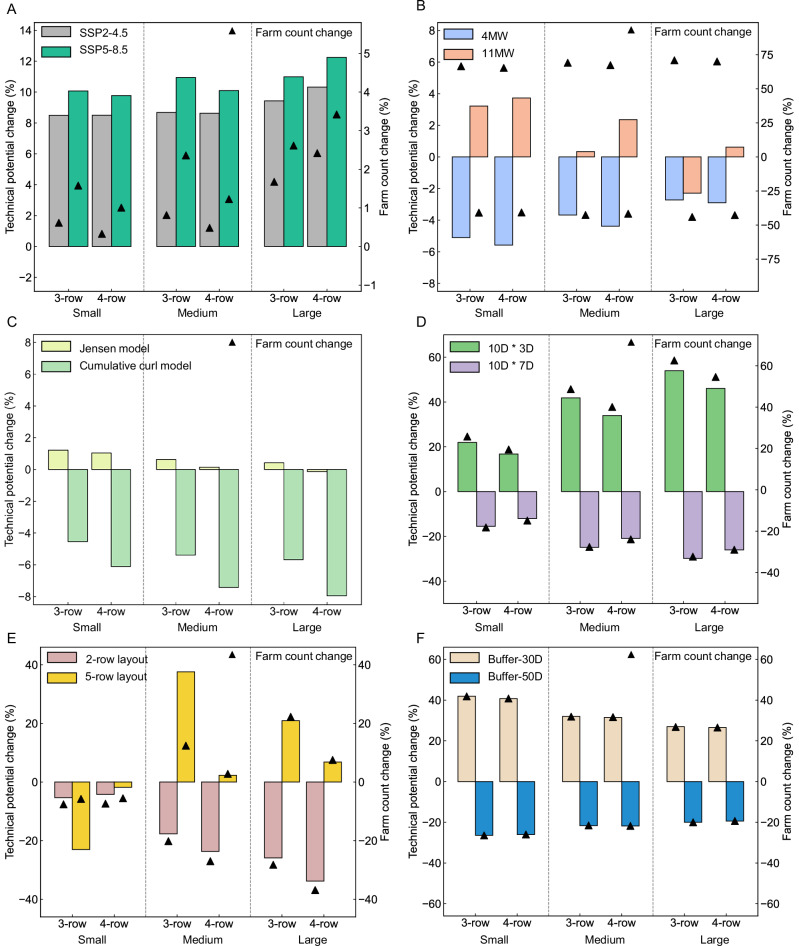
Impacts of climate change, turbine size, wake modeling, turbine spacing, and inter-farm buffer zones on offshore wind potential in China, compared to the baseline scenario, which assumes an 8 MW wind turbine, a Gaussian velocity model, and a *10D* * *5D* spacing rule. **A**–**C** show the effects of different wind speed data, turbine power ratings, and wake models on power generation potential. **D**–**F** illustrate the effects of turbine spacing, wind farm layout, and buffer zones between wind farms on power generation potential.

To explore the relative changes in potential across different scenarios, we used the M-8S3G, M-8S4G, M-8M3G, M-8M4G, M-8L3G, and M-8L4G scenarios as benchmark cases. We analyzed how changes in three key parameters—wind speed data, turbine size, and wake effect methods affect power generation potential and the number of wind farms. The results are shown in Fig. 8A–C, where all comparison scenarios correspond to the wind farm scale and layout indicated by the scenario labels in Fig. 2A.

As seen in Fig. 8A, compared to the MERRA-2 wind speed dataset, the potential under future wind speed scenarios increases for all configurations. The increase in wind farm numbers is proportionally lower than the increase in potential, suggesting that the potential increase is primarily due to higher wind speeds in the region. Additionally, the SSP5-8.5 wind speed scenario shows a slightly greater increase in potential compared to SSP2-4.5. Figure 8B illustrates the analysis of using turbines with different rated powers. It is clear that the total potential decreases when using 4 MW turbines, although the reduction is within 10%. However, the number of wind farms increases by more than 50%, primarily because the rotor diameter of the 4 MW turbine is significantly smaller than that of the standard 8 MW turbine (see Table S6), allowing for more wind farms. Nonetheless, the overall power generation decreases because each wind farm produces less energy. In contrast, scenarios using 11 MW turbines show increased potential in all configurations, except for the large 3-row wind farm (M-11L3G), where there is a slight potential decrease. The potential increase ranges from 0.34% to 3.7%, while the number of wind farms decreases by over 40%, indicating a higher single-farm generation potential for this turbine type.

To assess the impact of different wake models on the results, we compared the Jensen model and the cumulative curl model. As shown in Fig. 8C, compared to the Gaussian velocity model used in the six benchmark scenarios, the potential remains relatively unchanged with the Jensen model, with most scenarios showing a slight increase. In contrast, the cumulative curl model reduces the wind power potential across all scenarios, with decreases ranging from 4.5% to 7.9%.

Turbine spacing can have a significant impact on national generation potential. To assess this, we adjusted the row spacing (perpendicular to the prevailing downwind direction) in the baseline scenario from 5D to 3D and 7D. As shown in Fig. 8D, the generation potential of all wind farm configurations increases in the *10D* * *3D* scenario, and the potential gain becomes more pronounced as the size of the wind farm increases. When considering the row layout, the 3-row layout shows a slightly larger increase in potential compared to the 4-row layout of the same size. For the *10D* * *7D* scenario, the change in potential shows a relatively consistent trend, with all wind farm configurations showing a decrease in potential, and the decrease in potential becomes more pronounced as the size of the wind farms increases, but the absolute value of the decrease is smaller than that of the *10D* * *3D* scenario. In terms of the number of wind farms, the *10D* * *3D* scenario has a significant increase in generation potential, but its corresponding increase in the number of wind farms that need to be constructed is greater than the increase in potential, showing more tail losses and a corresponding decrease in economics. The *10D* * *7D* scenario, on the other hand, reduces the number of wind farms by a larger proportion than the reduction in potential and shows a higher single-farm generation potential.

In addition to extracting 3-row and 4-row layouts from remote sensing data, we also introduced 2-row and 5-row layouts to examine the impact of wind farm configurations on overall potential. As shown in Fig. 8E, for the 2-row layout, potential decreases in all scenarios, and the reduction becomes more pronounced as the wind farm size increases. In medium and large wind farms, the reduction is greater in the 4-row layout compared to the 3-row layout, except for small wind farms. From the perspective of wind farm numbers, the reduction in potential is positively correlated with the decrease in the number of wind farms. For scenarios with the 5-row layout, the potential change is more significant when switching from a 3-row layout than from a 4-row layout, with absolute changes exceeding 20%. Specifically, in small wind farms, the potential decreases by 25.9% when transitioning from a 3-row to a 5-row layout, while medium and large wind farms experience an increase of 37.6% and 20.9%, respectively. In contrast, when switching from a 4-row to a 5-row layout, the overall changes in power potential and wind farm numbers are relatively minor.

The wake behind the upstream wind farms reduces the amount of energy available at the downstream location, which leads to a reduction in the amount of power generated by the downstream wind farms^37^, so we have put in place a certain buffer zone between the wind farms. We also analyzed the effect of buffer zones between wind farms by adjusting the buffer from 40D (forty times the turbine rotor diameter) to 30D and 50D. As seen in Fig. 8F, compared to the six benchmark scenarios, reducing the buffer to 30D significantly increases both power potential and the number of wind farms, while increasing the buffer to 50D results in reductions in both. The changes in potential and wind farm numbers closely align in both cases.

## Discussion

This study advances the assessment of China’s offshore wind power potential by incorporating remote-sensing-derived turbine spacing practices, thereby enhancing the accuracy of both potential and cost evaluations. This approach aligns our findings more closely with real-world projects, moving beyond the often-optimistic estimates of previous studies. Specifically, we estimate the potential ranges of China’s offshore wind as 2.61-3.74 PWh yr^−1^ under six different benchmark scenarios, while the potential across 36 additional scenarios ranges from 2.46 to 4.20 PWh yr^−1^, with a median of 3.3 PWh yr^−1^. Such results are lower than most previous estimates in the literature, even though we used relatively loose screening conditions and large turbine power. This reduction highlights the importance of integrating realistic operational and environmental factors when conducting resource assessments and calls for a shift from broader, top-down approaches to more detailed, site-specific evaluations^38^. Our estimates for offshore wind are broadly consistent with IRENA and NREL values^24,39^, reinforcing the credibility of our cost framework. We also explicitly capture the influence of wind farm scale on LCOE, showing that larger farms benefit from economies of scale in infrastructure use. Additionally, the analysis shows considerable cost disparities between nearshore bottom-fixed and deep-water floating wind farms, with floating farms exhibiting much higher energy density but facing economic challenges^40^. The economic viability of these projects, especially floating wind farms, remains uncertain without substantial cost reductions or further subsidies.

The incorporation of future climate scenarios further refined our understanding of the potential impacts of climate change on wind resources. Our study found that future wind patterns, as modeled under the IPCC’s SSP2-4.5 and SSP5-8.5 scenarios, show slight improvements in power generation compared to the baseline data, suggesting that wind resource assessments should remain adaptable to ongoing changes in climate models and data. Additionally, the study quantified wake losses, which were found to be considerably higher than the typically cited 10%, indicating the need for more sophisticated wake loss models^41^. This finding underscores the complexity of accurately predicting energy production from wind farms, as wake effects vary significantly depending on factors like turbine placement and atmospheric conditions^41,42^. In addition, the spacing standards of wind turbines, the arrangement of wind farms, etc., will greatly affect the wake losses and overall potential, highlighting the importance of effective planning before construction^43^.

Despite the many advances presented in this study, there are still areas that require further research and improvement. While our methodology provides insights into national-scale variations across different scenarios and configurations, offering valuable references for future decisions on wind farm scale and turbine spacing, it simplifies certain aspects of offshore wind farm development. In practice, wind farms exhibit diverse layouts tailored to specific site conditions rather than adhering to a single configuration. This simplification overlooks the complexities of integrating real-world geographic constraints and atmospheric conditions to design site-specific layouts and scales. Future research should focus on optimizing spatial utilization and resource allocation by achieving a balance between energy output and economic efficiency under local constraints^44–47^. Additionally, improved modeling of intra- and inter-farm wake effects is crucial to better capture the complex interactions among turbines, particularly in large wind farms with diverse layouts^48,49^. Higher-resolution data is also essential for refining the study, allowing for more accurate assessments of environmental impacts and energy output^50,51^.

Furthermore, the global expansion of offshore wind energy provides valuable reference points for China’s development trajectory. The United Kingdom has achieved rapid deployment through centralized spatial planning and competitive auction schemes, while Germany emphasizes integrated grid development and environmental protection^52,53^. Denmark’s stable policies and public-private partnerships have driven cost reductions and social acceptance^54^. The United States is rapidly scaling up offshore wind development along its Atlantic coast while exploring floating technologies for Pacific waters^55^, Japan is pioneering floating wind technologies for deep-water applications^56^. While floating wind farms promise higher energy density, their high development costs present a significant challenge. Exploring ways to enhance the feasibility of deep-water installations and reduce costs for floating turbines is a critical area for future innovation^57^. As climate change continues to alter wind patterns and sea conditions, the design of offshore wind farms must adapt to these dynamic environmental changes^43,58^. This requires flexible strategies and designs that are resilient to evolving conditions^18,59^. The future development of offshore wind power in China needs to focus on several key aspects, including technological innovation, cost reduction, grid integration, and energy storage^15,58,60–62^. At the same time, it is crucial to ensure that offshore wind development aligns with environmental and social sustainability goals^63^, minimizing the ecological footprint of wind farms and fostering harmonious integration with local economies and communities^61^.

## Methods

### Offshore wind farm sites selection

The wind resource data used in this study include the MERRA-2 dataset, developed by NASA’s Goddard Space Flight Center. This dataset offers high-resolution climate simulations and meteorological observations intended to provide a continuous global record for climate research and applications. MERRA-2 covers 44 years (1979–2023) with an hourly temporal resolution and a spatial resolution of 0.5° latitude by 0.67° longitude (approximately 56 km by 61 km at mid-latitudes). In our analysis, we assumed a wind farm lifetime of 25 years, so we used the most recent 25 years of data (1999–2023)^26^. Additionally, we employed the Bias-corrected CMIP6 global dataset for dynamic downscaling of historical and future climate (1979–2100)^25^. This dataset is based on the 18 models from the CMIP6 and the ERA5 dataset from the European Center for Medium-Range Weather Forecasts (ECMWF),using the MVT method that corrects for mean, variance, and non-linear trend biases relative to ERA5. This correction enhances the credibility of climate-forced wind resource estimates used in our technical and economic analysis. Specifically, it provides a bias-corrected global dataset spanning both historical periods and future scenarios (SSP2-4.5 and SSP5-8.5), with a horizontal grid spacing of 1.25° × 1.25° and data recorded every six hours. We assumed a 25-year lifecycle for the wind farms and used data projected for the next 25 years (2025–2049). To identify suitable areas within China’s EEZ for wind farm placement, we filtered regions based on various factors such as water depth, wave height, protected areas, and distance from the shore. The data sources for these criteria are detailed in Table S2.

To establish spacing standards for existing offshore wind farms in China, we used the Global Offshore Wind Turbine (OWT) dataset^64^. This dataset derives from Sentinel-1 Synthetic Aperture Radar (SAR) time-series images. It employs percentile-based annual SAR image acquisition reduction and an adaptive thresholding algorithm on the Google Earth Engine platform to identify the spatiotemporal distribution of OWTs globally, with continuous updates. We extracted data specific to Chinese wind farms and manually identified well-organized wind farms. Additionally, we calibrated this information using data from 4Coffshore^65^ and DeepOWT^66^. We calculated the windward and vertical spacing of turbines within each wind farm and standardized these distances by the rotor diameter of each turbine (see Fig. S1), and finally selected a spacing standard of *10D* * *5D*. By analyzing the distribution of the number of rows in current wind farm layouts, we extracted the most common 3-row and 4-row layouts. Based on wind farm data and integrating the current capacity sizes of new wind turbines from IRENA^24^, we identified quintiles, weighted averages, and 95th percentiles of typical wind farm sizes and turbine types. Combining wind farm size, turbine capacity, layout pattern, and spacing standards, we defined 18 different types of wind farms (Arrangement of wind farm setting in Table S5).

During the placement process, the primary orientation of the wind farms is aligned with the prevailing wind direction, and multiple optimization iterations are conducted to maximize the number of wind farms that can be accommodated. A 40 * D buffer zone is included between the wind farms to minimize the impact of wake effects between them.

### Theoretical offshore wind power generation

For the calculation of potential, we conducted assessments at the farm scale, fully accounting for wake effect losses. We extrapolated the wind speed to the height of the selected turbines using the following formula^67–69^:

1 $$v={v}_{0}\times {\left(\frac{h}{{h}_{0}}\right)}^{\alpha }$$

where *v* is the extrapolated wind speed, ${v}_{0}$ is the initial wind speed, $h$ is the turbine height, ${h}_{0}$ is the reference height of the wind speed data, and *a* is the extrapolation coefficient calculated as follows:

2 $$\alpha=0.096*{{\mathrm{lg}}}{z}_{0}+{0.016*({{\mathrm{lg}}}{z}_{0})}^{2}+0.24$$

where *z*_0_ is the surface roughness set to 0.002^69,70^. The pressure law is used to correct the bias-corrected global dataset, assuming that 0.65 K 100 m^−1^ is reduced to obtain the corresponding height wind speed^71^.

This paper utilizes the Gaussian model, Jensen model, and Cumulative Curl model to calculate wake losses. The Gaussian model, used in the baseline scenario, is described in detail below, while the other two models are discussed in the relevant literature^33,34^. Gaussian model describes the velocity deficit as a Gaussian distribution in the spanwise direction.

3 $$\frac{\Delta v}{{v}_{\infty }}=\left(1-\sqrt{1-\frac{{C}_{{{{\rm{T}}}}}}{8{\left(\frac{{k}^{*}x}{{{{\rm{D}}}}}+\varepsilon \right)}^{2}}}\right)\times exp \left(-\frac{1}{2{\left(\frac{{k}^{*}x}{{{{\rm{D}}}}}+\varepsilon \right)}^{2}}\left\{{\left(\frac{z-h}{D}\right)}^{2}+{\left(\frac{y}{D}\right)}^{2}\right\}\right)$$

where *x*, *y*, and *z* are streamwise, spanwise, and vertical coordinates, respectively, *D* is the wind turbine diameter, and *h* is the hub height level. ${C}_{{{{\rm{T}}}}}$ is the thrust coefficient, which is calculated in this study by importing the power curve of the turbine into windPRO^72^. *k*^*∗*^ denotes the wake growth rate, which is a function of thrust coefficient and local streamwise turbulence intensity^73^. ε is given by the following equation:

4 $$\varepsilon=0.2*\sqrt{\frac{1}{2}\frac{1+\sqrt{1-{C}_{{{{\rm{T}}}}}}}{\sqrt{1-{C}_{{{{\rm{T}}}}}}}},{C}_{{{{\rm{T}}}}} < 0.9$$

A wind turbine within a wind farm is influenced by multiple upstream turbines. To account for the combined effect of their cumulative wakes, we apply the principle of superposition, defined as follows:

5 $${v}_{i}={v}_{\infty }-{\sum }_{k}\left({v}_{\infty }-{v}_{{ki}}\right)$$

where ${v}_{i}$ is the velocity at the turbine $i$ and ${v}_{{ki}}$ is the wake velocity of the turbine $k$ at turbine $i$, considering only those turbines whose wakes interact with turbine $i$.

The power of a wind turbine is given by the turbine parameters and the interpolation function:

6 $$P\left(\nu \right)=Pr\cdot \left\{\begin{array}{c}\begin{array}{cc}0\hfill & \nu < {\nu }_{c}\hfill\\ {Interpolated}\hfill & {\nu }_{c}\le \nu \le {\nu }_{r}\end{array}\\ \begin{array}{cc}1\quad\qquad\qquad & {\nu }_{r}\le \nu \le {\nu }_{f}\\ 0\hfill & \nu \ge {\nu }_{f}\hfill\end{array}\end{array}\right.$$

where $Pr$ is the rated power of the turbine, ${\nu }_{c},{\nu }_{r},{\nu }_{f}$ are the cut-in wind speed, rated wind speed, and cut-out wind speed, respectively. The interpolation function is constructed from the discrete wind speed and power output pairs, allowing for estimation of the turbine’s power at any wind speed within the operational range. The turbine power curves and key parameters used in this study are presented in Fig. S12, Table S6. The annual energy production (*AEP*) of the wind farm is:

7 $${AEP}={\sum }_{k=1}^{N} {\sum }_{i=1}^{n}P({v}_{i})\times f({v}_{i})\times T$$

where *N* the number of turbines in the wind farm, $n$ is the number of different wind speeds, and $T$ represents the total number of hours in a year, $P({v}_{i})$ is the power output at wind speed ${v}_{i}$, $f({v}_{i})$ is the frequency or probability of wind speed ${v}_{i}$. We then combined the *AEP*s of many years to obtain the average *AEP*.

### Calculation of costs

The specific levelized cost of electricity (*LCOE*) methodology used in this analysis is based on a modification of Tyler et al.^39^ in the Handbook of Techno-Economic Assessment of Energy Efficiency and Renewable Energy Technologies^74^, with the following equation:

8 $${{LCOE}\left(D,d\right)}_{i}=\frac{\left({C{ape}Ex}_{i} * {FCR}\right)+{O{pEx}}_{i}}{\left(\frac{{{AEP}}_{i}}{1,000}\right)}$$

where ${{LCOE}}_{i}$ represents the levelized cost of energy at each wind farm, measured in dollars per megawatt-hour ([USD/MWh]); ${{CapeEx}}_{i}$ denotes the capital expenditures, which are the initial costs of constructing the wind farm, measured in dollars per kilowatt (USD/kW); FCR is the fixed charge rate, a percentage that accounts for the cost of capital and financing over the life of the wind farm; ${{OpEx}}_{i}$ signifies the operational expenditures at each wind farm, including maintenance and operational costs, measured in dollars per kilowatt per year (USD/kW/yr); and ${{AEP}}_{i}$ stands for the net average annual energy production at each wind farm, measured in megawatt-hours per megawatt per year (MWh/MW/yr). This formula integrates both the upfront and ongoing costs with the energy output to determine the cost-effectiveness of the wind farm over its operational lifetime.

The fixed charge rate is the revenue per amount of investment required to cover the investment cost, including interest paid on debt and return on equity, so that ${CapeEx}\times {FCR}$ is the effective annuity payment and is constant each year. In this study, the *FCR* is calculated as the product of the Capital Recovery Factor (*CRF*) and a Project Finance Factor (*PFF*) that accounts for additional financing costs such as fees, contingencies, and reserves^39^.

9 $${FCR}={CRF}\times {PFF}$$

10 $${CRF}=\frac{{WACC}}{1-{\left({WACC}+1\right)}^{-n}}$$

Here, *PFF* = 1.05, consistent with NREL guidelines and WACC is the real weighted average cost of capital, and *n* = 25 years is the assumed project operational life. Based on China’s wind financial data, we assume a real WACC of 2.84%^75^. Considering that the water depth and offshore distance during the construction of offshore wind farms seriously affect the capital expenditure of wind farms, we constructed a costing model about water depth and offshore distance^76–78^. The corresponding capital expenditure is calculated as follows:

11 $$C{apE}{x}_{i}=	 {\sum }_{j=1}^{N}({C}_{{turb}}(\Pr )_{j}+{C}_{{found}}{(d)}_{j}+{C}_{{trans}}{(D)}_{j}+{C}_{{inst}}{(D,d)}_{j}\\ 	+{C}_{{project}}{\left(D,d\right)}_{j}+{C}_{{soft} \_{capex}}{\left(D,d\right)}_{j})\,$$

The cost components associated with offshore wind farms can be described as follows: ${C}_{{turb}}$ refers to the turbine cost, which is dependent on the capacity of the wind farm. ${C}_{{foun}}$ denotes the infrastructure cost, which varies with water depth $\left(d\right)$, affecting the foundation construction complexity. ${C}_{{trans}}$ accounts for transmission costs, which depend on the distance $\left(D\right)$ to the nearest shoreline, reflecting the expense of connecting the wind farm to the power grid. ${C}_{{inst}}$ is the installation cost, which increases with distance $\left(D\right)$, covering logistical and operational expenses. ${C}_{{project}}$ represents the project development cost, covering preliminary expenses such as planning and permitting. ${C}_{{soft}\_{capex}}{\left(D,d\right)}_{j}$ includes construction financing, insurance, commissioning, contingencies, and decommissioning, calculated using industry-standard factors$.$ The water depth $\left(d\right)$ impacts the infrastructure costs due to the added complexity of constructing foundations in deeper waters. The distance to the nearest shoreline $\left(D\right)$ influences both transmission and installation costs, as remote locations require more extensive resources.

The offshore distance is also introduced as a variable in the calculation of the O&M cost of offshore wind farms, which is calculated as follows:

12 $${{OpEx}}_{i}=\left\{\begin{array}{cc}{C}_{{Bottom}-{fixed}}(d),& d\le 60\\ {C}_{{Floating}}(d),\hfill & d > 60\end{array}\right.$$

here$\,{C}_{{Bottom}-{fixed}}$ represents the variation of O&M cost with offshore distance d for fixed offshore wind, and ${C}_{{Floating}}$ represents the variation of O&M cost with offshore distance d for floating offshore wind. d. For details, see Supporting Information.

## Supplementary information

Supplementary Information

Transparent Peer Review file

## Source data

Source Data

## Data Availability

The main data supporting the findings of this study are available within the paper and Supporting Information; other data can be requested from the author upon request. Furthermore, datasets are openly accessible via the online repository at 10.6084/m9.figshare.29625785. Source data is available as a Source Data file. Source data are provided with this paper.

## Code availability

The full set of code employed for data processing, quantitative analysis, and figure generation in this study is publicly available in the online repository at 10.6084/m9.figshare.29625785. The repository also includes example datasets and comprehensive documentation detailing the structure and usage of the code.
