# Supplementary material for: Substantially lower estimates in China’s offshore wind potential using farm-scale spatial modeling and wake effects
Source: Nat Commun. 2026 Jan 26;17:2043. doi: 10.1038/s41467-026-68655-2 (PMC12946245; doi:10.1038/s41467-026-68655-2)
Supplement: Supplementary file 1 — Supplementary Information [file 41467_2026_68655_MOESM1_ESM.pdf]

Supporting Information for

Substantially Lower Estimates in China's Offshore Wind Potential Using Farm-Scale Spatial Modeling and Wake Effects

Shiwei Xu, Gege Yin, Peiyu Hu, Di Dong, Yue Qin, Yu Liu, Gang Liu, Lili Song, Chuan Zhang*

*Corresponding author: Chuan Zhang, czhang@pku.edu.cn

This PDF file includes:

Supporting text
Figs. S1 to S13
Tables S1 to S7
SI References

Supplementary Text

1. Wind farm data analysis

We first used a clustering algorithm to merge independent turbines into wind farms for the obtained wind turbine dataset, and then corrected it with other datasets (DeepOWT [1] and 4C Offshore [2]) and removed obvious abnormal turbines and booster stations. We further screened the obtained wind farms to obtain wind farms with relatively regular shapes. Combined with the wind data of the wind farm location (Global Wind Atlas [3]), we calculated the geographical distance of each farm along the main wind direction and perpendicular to the main wind direction, and standardized the distance by the turbine rotor diameter data of each farm(Fig. S3.). At the same time, we obtained the most commonly used layout of wind farms through a semi-supervised method, and combined the calculated number of turbines for each wind farm with the IRENA report data [4] to obtain a typical wind farm layout. The flowchart of this section is shown in Fig. S4.

2. Theoretical offshore wind power generation

We have already introduced the main contents of power generation calculation in the main text, and here we will further explain the wake calculation method.

Given a wind farm with *N* wind turbines and a certain layout pattern where superscript o indicates original (unrotated) coordinate system (equation (1)). Suppose a single direction wind has a speed of v_0 with direction θ , the wind speed at each turbine *i* is denoted as v_i where v_0 and v_i wind direction is defined as the angle rotated clockwise from negative y axis direction as illustrated in Fig. S5.

$$layout = \begin{bmatrix} x_1^0 & x_2^0 & \cdots & x_N^0 \\ y_1^0 & y_2^0 & \cdots & y_N^0 \end{bmatrix} \tag{1}$$

If the wind direction rotates clockwise, in order to ensure that the wind direction is the same as the negative direction of the y axis, we can rotate the x, y axis clockwise by an angle. The new coordinates in the rotated space can be calculated by the following equation:

$$\begin{bmatrix} x' \\ y' \end{bmatrix} = \begin{bmatrix} \cos(\theta) & -\sin(\theta) \\ \sin(\theta) & \cos(\theta) \end{bmatrix} \begin{bmatrix} x \\ y \end{bmatrix} \tag{2}$$

x and y are the positions of the wind turbine in the original coordinate system, x' and y' are the new coordinates after rotation, θ is the rotation angle. Then we used Gaussian model [5,6], Jensen model [7], Cumulative Curl model [8,9] to calculate the wake loss of each wind farm.

3. Calculation of costs.

Similarly, we introduced the main methods and key formulas of cost calculation in the main text. Here we further explain each component. We divide capex costs into turbine costs, infrastructure costs, installation costs, transmission costs, and development costs. Turbine costs are related to the rated power of the wind turbine. Infrastructure costs, installation costs, transmission costs, etc. are determined by the water depth and offshore distance to determine the corresponding technical selection and then calculated using different formulas. Development costs and decommissioning costs are calculated based on the percentage of the total of other investment costs of the project [10–15]. Additionally, we incorporate soft CAPEX elements following NREL's ORBIT methodology, including construction financing, insurance, commissioning, and contingencies[16]. Unless otherwise specified, the cost unit is USD/MW, d represents water depth, D represents distance from shore, both unit is m. Here, *D* is calculated as the shortest distance from each turbine

location to the nearest shoreline and is used as a proxy for both port distance (for installation cost estimation) and transmission distance (for grid connection cost estimation). We acknowledge that this simplification may not fully represent real-world infrastructure layouts involving predefined ports or Points of Interconnection (POIs), but it provides a practical approximation for large-scale analysis. The specific contents are as follows:

a. CAPEX calculations

Wind Turbine: The cost of a wind turbine is usually expressed as a function of the rated power, assuming that it does not depend on the type of installation. The cost (M$/MW) of the wind turbine used in the study is adapted from Maienza et al.[12] and updated with recent market cost factors. The specific formula used is as follows:

$$C_{turb} = (1.6 \cdot p_T - 1.9) \cdot 1.1789 / p_T \tag{3}$$

where p_T is the installed power (MW) of one turbine.

Foundation Cost: The foundation cost is significantly influenced by the water depth at the planned wind farm location, as the cost and capacity of different technologies vary greatly across various water depth ranges. In this study, Monopile and Jacket are chosen as the foundation types for fixed wind turbines, while WindFloat is selected for floating wind turbines based on the relationship between water depth and cost. The cost formulas are based on Myhr et al.[14], Bjerkseter[17], with WindFloat incorporating modules such as anchors, mooring lines, chains, and substructures[12], as detailed below:

Monopile (d ≤ 25)：

$$C_{\text{found}}(d) = 156.579d^2 + 477.2811d + 320140.16 \tag{4}$$

Jacket (25 < d ≤ 60):

$$C_{\text{found}}(d) = 88.96496d^2 - 1767.33d + 413805.02 \tag{5}$$

WindFloat (d > 60):

$$C_{found}(d) = 1283881 + 133d \tag{6}$$

Transmission costs：Submarine transmission cables are relatively costly, and the optimal choice of technology depends significantly on the cost of all transmission infrastructure required for different transmission distances and the capacity class of the connected projects. We select the type of transmission line for the wind farm based on the transmission distance: for distances within 56 km, we use alternating current (AC) transmission lines; for distances beyond 56 km, we use high voltage direct current (HVDC) transmission lines. Transmission costs are divided into export cables (connecting wind farm to shore) and internal cables (connecting turbines within the farm).The cost of the export cable C_{export} (in USD) is modeled as a function of the export distance D (in meters), the main cable length within the farm L_{main} (in km), and the total farm capacity C_{total} (in MW). The functional form is given by:

For HVAC (D ≤ 56000):

$$C_{export}(D, L_{main}) = 7{,}598.31 \times \left(\frac{D}{1000} + \frac{L_{main}}{C_{total}}\right) + 50{,}750.81 \tag{7}$$

For HVDC (D > 56000):

$$C_{export}(D, L_{main}) = 1{,}966.62 \times \left(\frac{D}{1000} + \frac{L_{main}}{C_{total}}\right) + 346{,}667.61 \tag{8}$$

The internal array cable cost C_{internal} is calculated based on the internal cable length L_{internal} (in km), also normalized by farm capacity:

$$C_{internal}(L_{internal}, C_{total}) = \frac{266388 \times L_{internal}}{C_{total}} \tag{9}$$

The cable lengths L_{internal} and L_{main} are determined from typical wind farm layouts. We adopt the "Grid layout with no partial strings" configuration as presented in the ORBIT reference model[16], with representative 2D arrangements illustrated in Fig. S13. Cable lengths for different layout types and turbine models are summarized in Table S7, which includes values for 3-row and 4- row configurations across small (SWT 4 130), medium (V164_8), and large (EW_11_208) turbines.

Installation Cost: The installation of wind farms encompasses the installation of wind turbines, transmission lines, and wind turbine substructures, with installation costs varying for each type of underlying technology. For all technologies, the cost differences related to offshore distance are influenced by the time required for ships to reach the offshore facility and the daily costs of personnel. Meanwhile, costs related to water depth are reflected in the different turbine installation types. The costs have been adjusted for inflation and cost deflation effects over time. The cost results for the three turbine installation types, after considering these factors, are presented below[12,14,17]:

Monopile (d ≤ 25):

$$C_{\text{inst}}(d, D, L_{\text{internal}}, L_{\text{main}}, C_{\text{total}}) = (C_{\text{wt\_inst}} + C_{\text{sub\_inst}} + C_{\text{export\_inst}} + C_{\text{internal\_inst}}) \times 1.33 \times 1.24 \tag{10}$$

Jacket (25 < d ≤ 60):

$$C_{\text{inst}}(d, D, L_{\text{internal}}, L_{\text{main}}, C_{\text{total}}) = (C_{\text{wt\_inst}} + C_{\text{sub\_inst}} + C_{\text{export\_inst}} + C_{\text{internal\_inst}}) \times 1.33 \times 1.24 \tag{11}$$

WindFloat (d > 60):

$$C_{\text{inst}}(d, D, L_{\text{internal}}, L_{\text{main}}, C_{\text{total}}) = (C_{\text{wt\_inst}} + C_{\text{sub\_inst}} + C_{\text{export\_inst}} + C_{\text{internal\_inst}} + C_{\text{mooring\_inst}}) \times 1.33 \times 1.24 \tag{12}$$

Where:

$$C_{\text{wt}_{\text{inst}}} = \begin{cases} 1.492 \times D & \text{(Monopile)} \\ 1.906 \times D & \text{(Jacket)} \\ 0.644 \times D & \text{(WindFloat)} \end{cases} \tag{13}$$

$$C_{\text{export\_inst}} = \frac{590000 \times \left(\frac{D}{1000} + L_{\text{main}}\right)}{C_{\text{total}}} \tag{14}$$

$$C_{\text{internal\_inst}} = \frac{196667 \times L_{\text{internal}}}{C_{\text{total}}} \tag{15}$$

$$C_{\text{mooring\_inst}} = 0.222 \times D \tag{16}$$

$C_{\text{sub\_inst}}$ is estimated to be around 42400 USD per MW[17].

Project development cost: Following the methodology of Martinez et al.[10] and the NREL ORBIT model[16], we define development costs to include expenses related to site auctions, site assessment plans, construction planning, installation planning, environmental surveys, metocean and geotechnical assessments, as well as overall project management. Drawing from these sources, and consistent with definitions used in

the "Development and Project Management" category of NREL's Cost of Wind Energy Review reports[15], we adopt a development cost estimate of $225000 USD/MW, adjusted to 2022 USD.

Soft CAPEX calculations: According to the NREL ORBIT methodology, Soft CAPEX includes construction financing, insurance, commissioning, contingencies, and decommissioning, calculated using industry-standard factors:

Construction Insurance:

$$C_{insurance} = 0.0115 \times \left(C_{turb} + C_{found} + C_{trans} + C_{project}\right) \quad (17)$$

This covers all-risk property, delays in start-up, third-party liability, and broker's fees.

Commissioning:

$$C_{commissioning} = 0.0115 \times \left(C_{turb} + C_{found} + C_{trans} + C_{project}\right) \quad (18)$$

Decommissioning:

$$C_{decommissioning} = 0.175 \times C_{inst} \quad (19)$$

Procurement Contingency:

$$C_{proc\_contingency} = 0.0575 \times \left(C_{turb} + C_{found} + C_{trans} + C_{project}\right) \quad (20)$$

Installation Contingency:

$$C_{inst\_contingency} = 0.0345 \times C_{inst} \quad (21)$$

Construction Financing:

Construction financing is calculated using a time-weighted financing factor based on the standard ORBIT expenditure schedule. The financing factor is calculated as:

$$F_{financing} = \sum_{k=0}^{5} S_k \times [1 + (1 - t) \times ((1 + r)^{k+0.5} - 1)] \quad (22)$$

where S_k is the spend fraction in period k (Year 0: 25%, Year 1: 25%, Year 2: 30%, Year 3: 10%, Year 4 : 10%,Year 5: O%), St= 0.26$ (tax rate), and $r = 0.044$ (interest rate during construction).

$$C_{financing} = \left(F_{financing} - 1\right) \times (C_{insurance} + C_{commissioning} + C_{decommissioning} + C_{proc_{contingency}} + C_{inst_{contingency}} + C_{turb} + C_{found} + C_{trans}) \quad (23)$$

b. Operation and maintenance costs

In this study, Operation and Maintenance (O&M) costs follow the methodology of Bosch et al.,[11] where O&M costs depend on the type of technology, which is determined by the water depth. Additionally, the costs vary significantly between fixed and floating turbines due to factors such as maintenance vessels and operating window periods. The following section specifically presents the O&M cost per MW as a function of distance used in this study:

Monopile and Jacket (d<60):

$$C(D) = (0.0212D + 37292) \cdot 1.2 \cdot 0.779 \quad (24)$$

WindFloat (d > 60):

$$C(D) = (0.0433D + 39191) \cdot 1.2 \cdot 0.779 \quad (25)$$

Supplementary figures

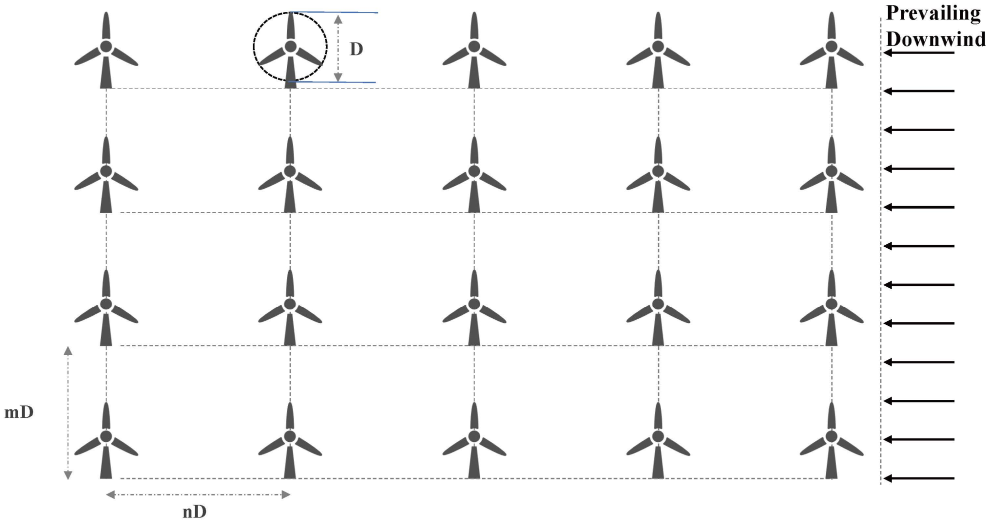

Fig. S1. Schematic diagram of wind turbine spacing rules. The wind turbines are evenly arranged on the available map, with different spacing standards for the prevailing downwind direction and vertical prevailing downwind direction.

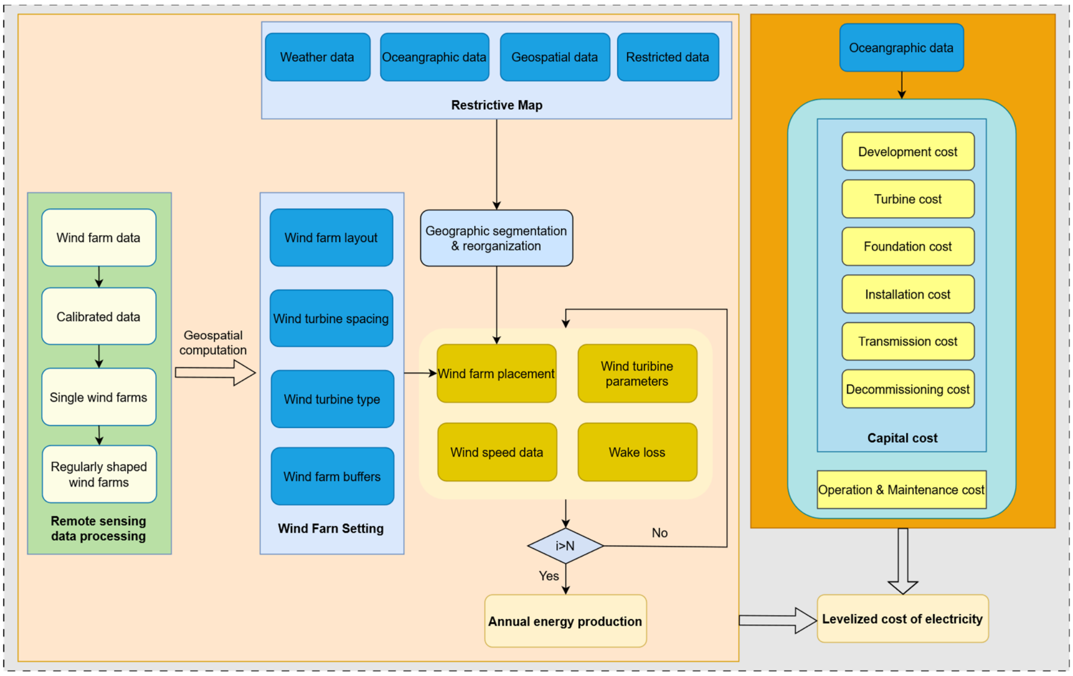

Fig. S2. A bottom-up framework for analyzing realistic offshore wind farms based on remote sensing data analysis. Remote sensing data of existing wind farms in China are processed to obtain the typical wind farm layout and spacing criteria, the wind farms are placed on the base map screened according to various siting criteria based on the wind direction and the maximization of wind farms that can be accommodated, and the annual power generation of each wind farm is calculated in conjunction with the wake loss model, and then the cost is calculated through the integrated economic model of offshore wind farms.

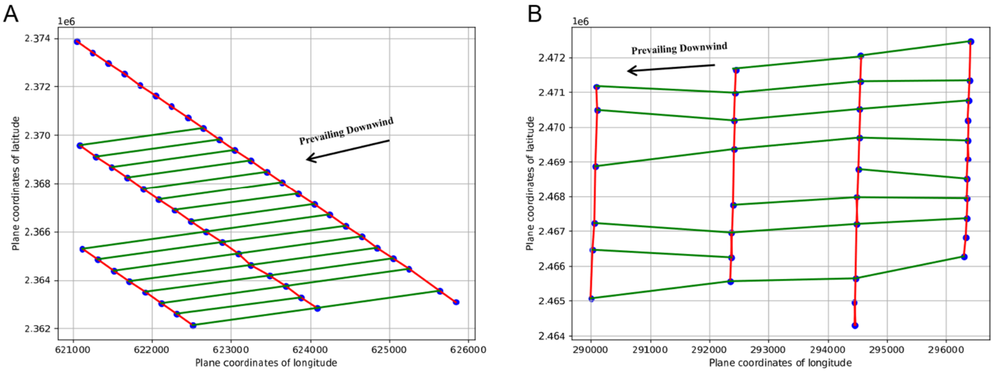

Fig. S3. Example of wind farm spacing calculation. (A) An example of a 3-row arrangement, which is called CECEP Yangjiang Nanpeng Island Offshore Wind Farm. **(B)** An example of a 4-row arrangement, which is CGN Huizhou Offshore Wind Farm. The blue dots in the picture represent wind turbines, the green line represents the direction of the main wind, and the red line represents the direction perpendicular to the main wind direction.

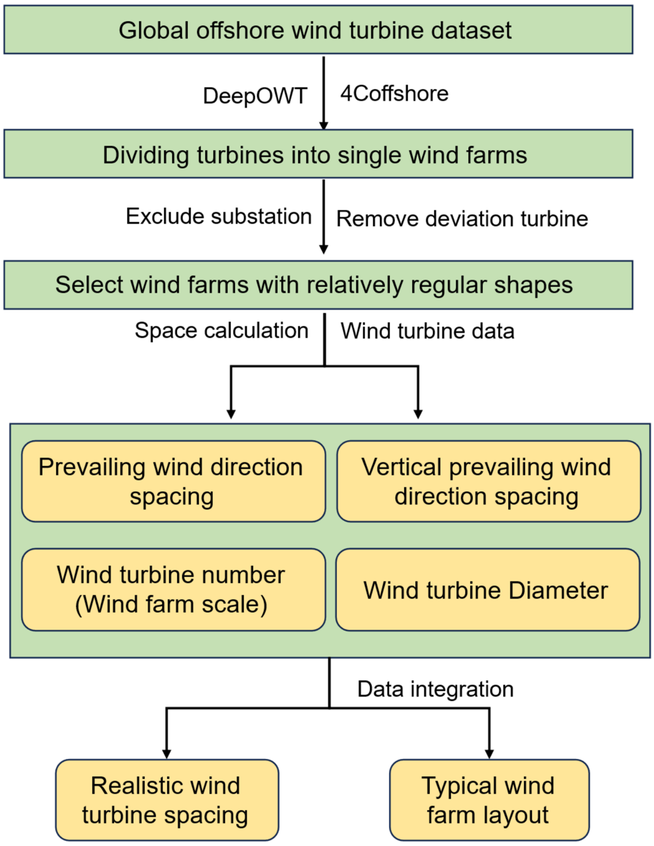

Fig. S4. Flowchart of processing wind farm remote sensing data set, including data correction, wind farm segmentation, wind farm screening, geographic distance calculation and other processes.

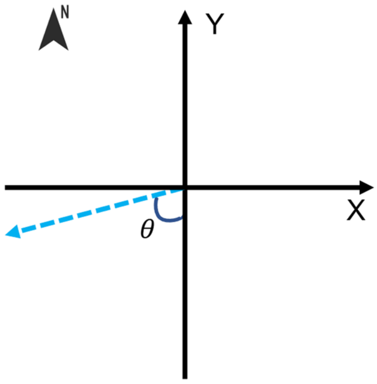

Fig. S5. Wind direction definition.

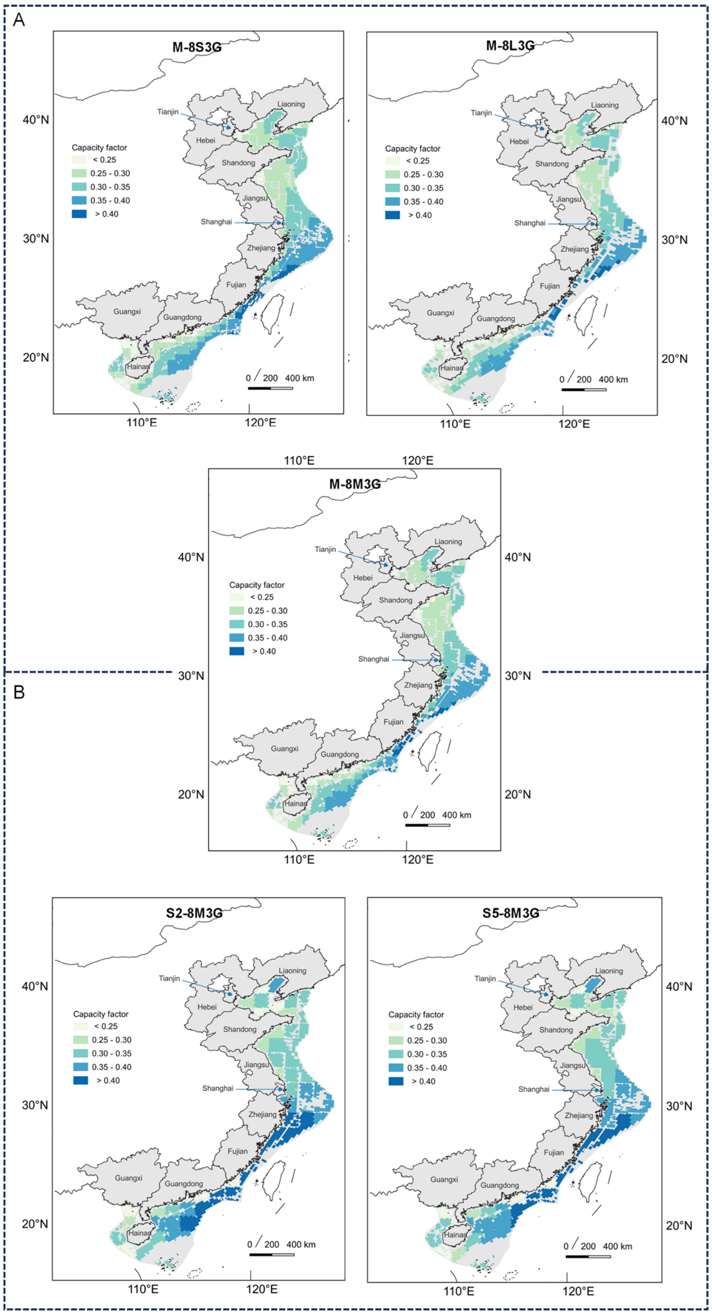

Fig. S6. Capacity factors of wind farms under different scenario (3-row layout). (A), M-8S3G and M-8L3G scenarios. (**B**), S2-8M3G and S5-8M3G scenarios. M-8M3G scenarios in the middle of the two diagrams. The capacity factor of each wind farm after taking into account the wake effect shows that relatively speaking, the farther offshore distance the higher the capacity factor, which is due to the better wind conditions in the deep and distant sea.

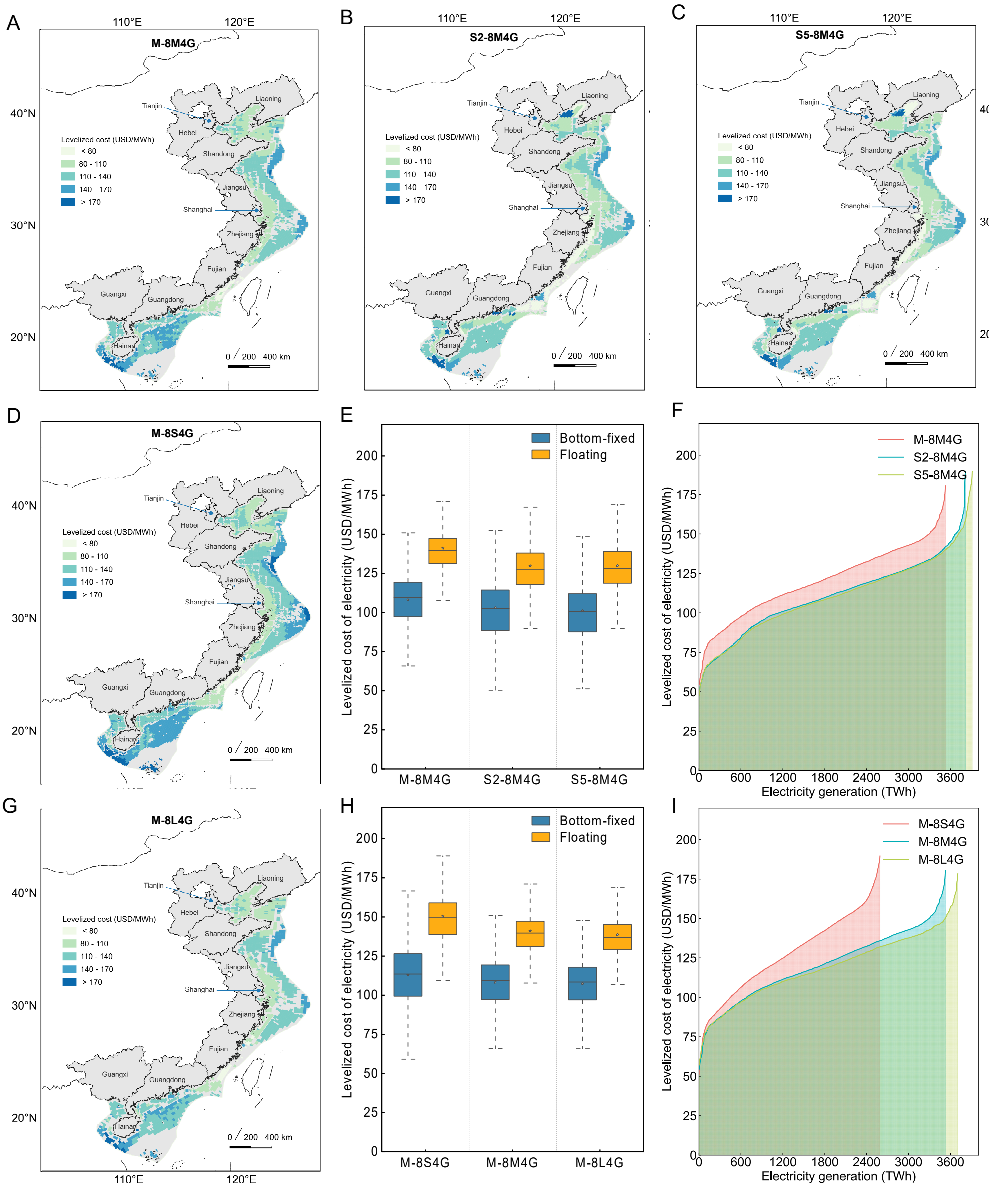

Fig. S7. Distribution of offshore wind farms and wind power supply curves in China. (**A** to **C**) Spatial distribution of LCOE for offshore wind power at the farm scale in China, with (**A**), (**B**), and (**C**) representing the M-8M4G, S2-8M4G, and S5-8M4G scenarios, respectively (8MW, 4-row layout with 60 turbines, gaussian model). (**D**), (**G**) Spatial distribution of LCOE for M-8S4G and M-8L4G, respectively. (**E**), Distribution of LCOE values across the three wind speed scenarios (M-8M4G, S2-8M4G, and S5-8M4G scenarios) at the farm scale. (**F**), National offshore wind supply curves for the M-8M4G, S2-8M4G, and S5-8M4G scenarios at the farm scale. (**H**), Distribution of LCOE values at the farm scale for three layout options (M-8M4G, M-8S4G and M-8L4G scenarios). (**I**), National offshore wind supply curves at the farm scale for three layout options M-8M4G, M-8S4G and M-8L4G scenarios).

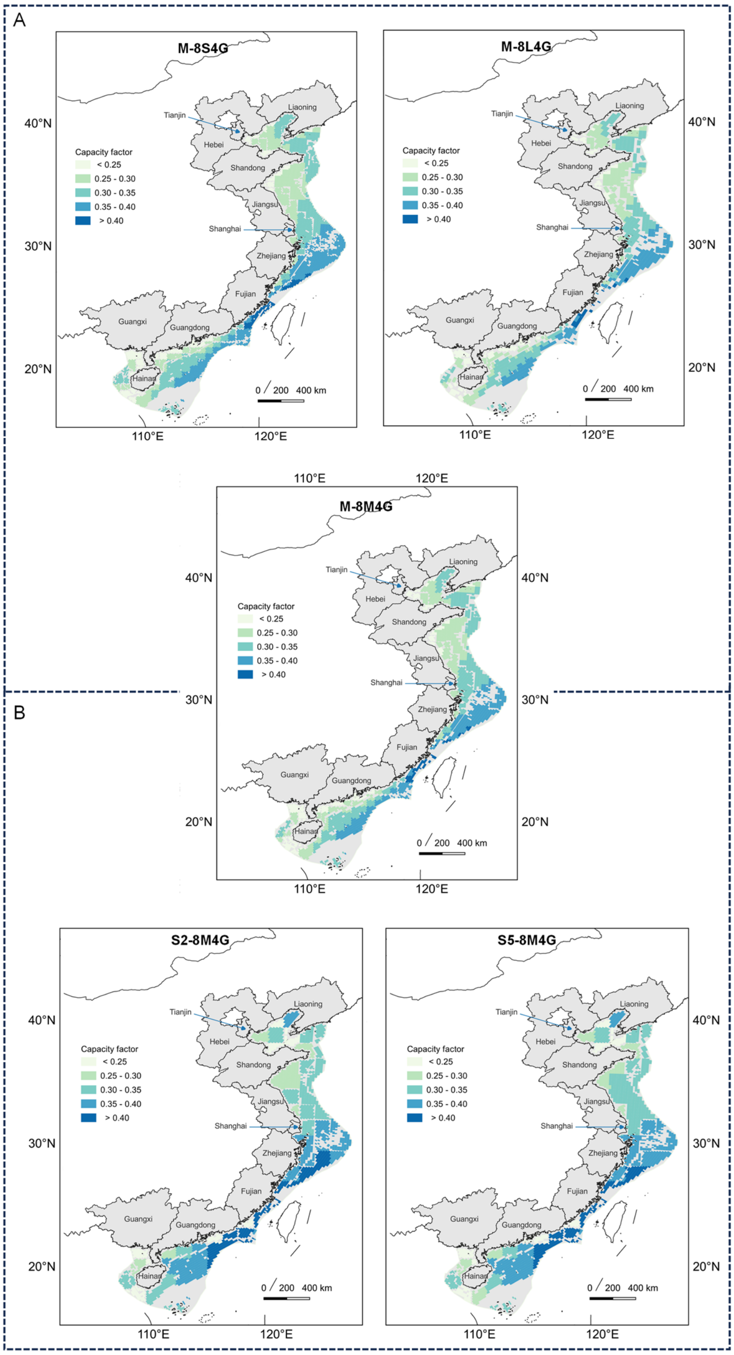

Fig. S8. Capacity factors of wind farms under different scenario (4-row layout). (A), M-8S4G and M-8L4G scenarios. (**B**), S2-8M4G and S5-8M4G scenarios. M-8M4G scenarios in the middle of the two diagrams. Similar to the 3-row layout, the capacity factor of the wind farms, after taking into account the wake effect, shows that, relatively speaking, the further the distance offshore, the higher the capacity factor, which is due to the better wind conditions in the deeper waters.

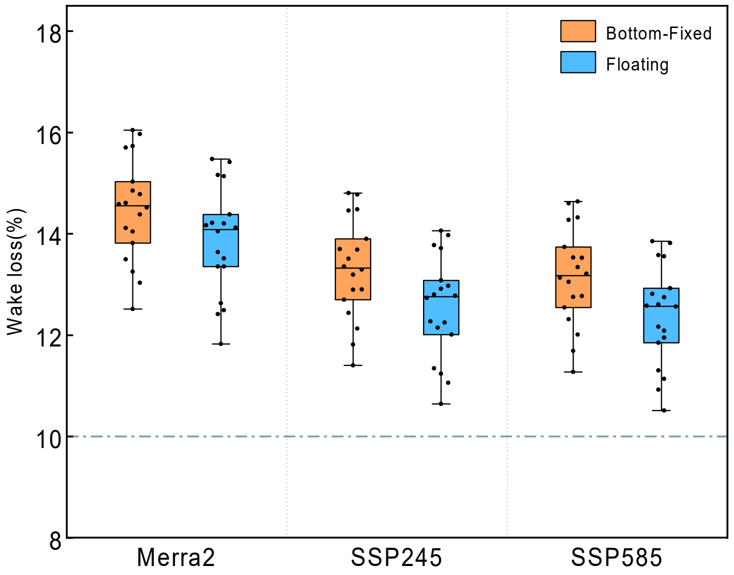

Fig. S9. Comparison of wake effects between fixed and floating wind farms under different wind speed dataset. For each wind speed dataset, there are 18 types of configurations, consisting of 4MW, 8MW, and 11MW turbine types, with 3-row and 4-row layouts, across three wind farm sizes: small, medium, and large, all using Gaussian wake modeling. Floating wind farms have lower wake losses than fixed wind farms, which is mainly due to the fact that floating wind farms have better wind conditions, and therefore higher power production even after wake attenuation.

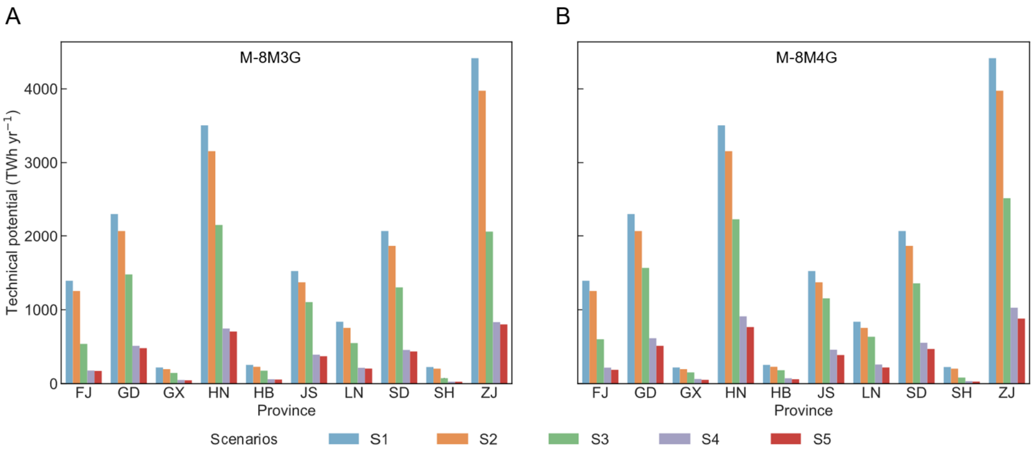

Fig. S10. Changes in offshore wind potential by province in the M-8M3G and M-8M4G Scenario. (A), M-8M3G scenario **(B)**, M-8M4G scenario.

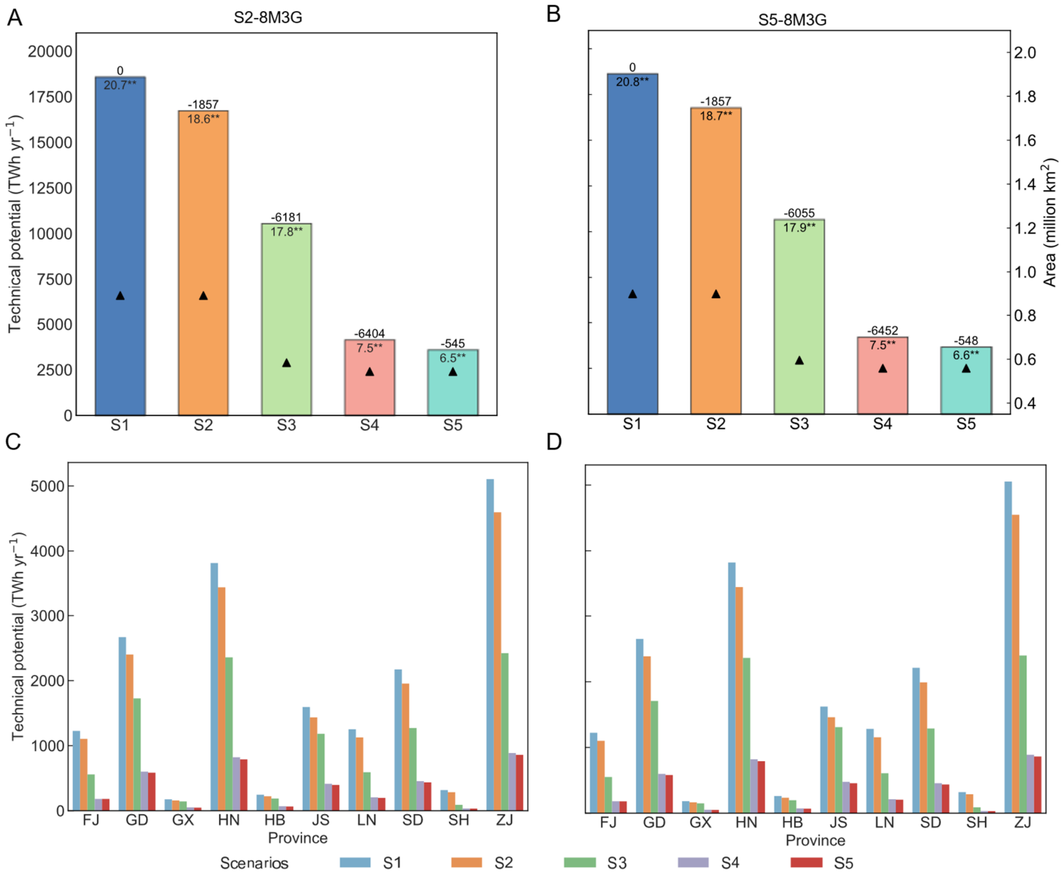

Fig. S11. Changes in power generation potential under different parameter adjustments in S2-8M3G and S5-8M3G scenarios. (A-B) Changes in overall national offshore wind potential. **(C-D)** Changes in offshore wind potential by province. S1, calculated using the unit area installed density method without considering wake losses; S2, calculated using the unit area installed density method with estimated wake losses (10%); S3, calculated at the farm scale without considering inter-farm buffer zones and estimating wake losses (10%); S4, calculated at the farm scale considering inter-farm buffer zones but without wake losses; and S5, calculated at the farm scale considering both inter-farm buffer zones and wake losses. The farm-scale calculations all use a 3-row layout, with a wind farm size of 60 units.

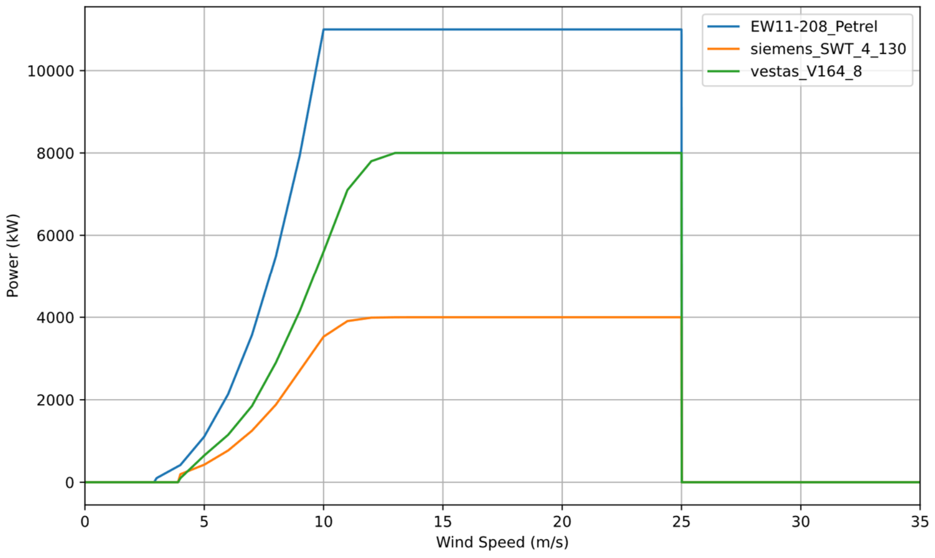

Fig. S12. The power curves of three types of wind turbines with rated powers of 4MW, 8MW and 11MW are used in this article. The specific parameters are shown in Table S6.

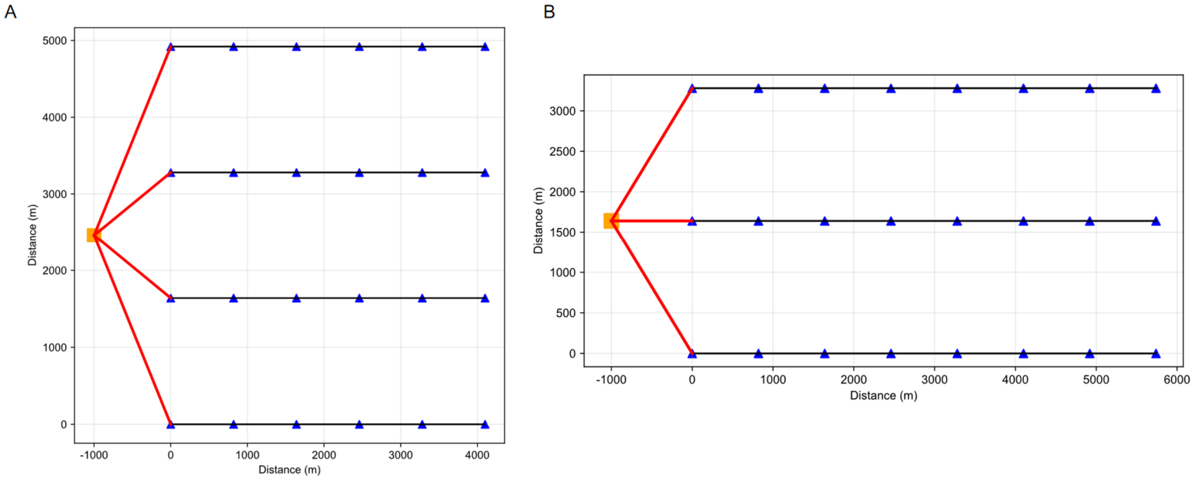

Fig. S13. Layout cable distribution. (A) 4-row, 6-column layout cable distribution**. (B)** 3-row, 8-column layout cable distribution.

Supplementary tables

Table S1. Offshore wind farm spacing data extracted from remote sensing data in China.

Wind farm	PWDS (m)	VPWDS (m)	Turbine Model	D (m)	PWDS/D	VPWDS/D
CECEP Yangjiang Nanpeng Island	1714	503	MySE5.5-155	155	3.2	11.1
CGN Huizhou	1782	1053	V174-9.5 MW	174	6.1	10.2
CGN Rudong Demonstration	1157	971	SWT-4.0-130	130	7.5	8.9
CGN Shanwei Houhu -West	2163	510	MySE5.5-155	155	3.3	14
CGN Yangjiang Nanpeng Island	1960	538	MySE5.5-155	155	3.5	12.6
CSIC Jiangsu Rudong H3-1 300MW	1623	790	H151-5MW	151	5.2	10.7
CTGNE Jiangsu Dafeng H8-2	1611	782	GW 155-4.5MW	155	5	10.4
CTGNE Yangjiang Shapa - phase I	2231	498	MySE5.5-155	155	3.2	14.4
CTGNE Yangjiang Shapa - phase II	2399	601	MySE6.45-180	180	3.3	13.3
CTGNE Yangjiang Shapa - phase V	1824	665	MySE6.45-180	180	3.7	10.1
Changle Area A	1604	661	DEW-D10000-185	185	3.6	8.7
Changle Area C - phase 2	1666	985	DEW-D10000-185	185	5.3	9
Dafeng H5	1484	1065	GW184-6.45MW	184	5.8	8.1
Dafeng H6	1790	1090	GW184-6.45MW	184	5.9	9.7
Datang Jiangsu Binhai 300MW -East	1227	736	MySE3.0-135	135	5.5	9.1
Donghai Bridge Offshore Wind Farm - phase II (Extension) project -North	1012	820	W3600-116	116	7.1	8.7
Dongtai Four (H2) 300MW -North	1524	681	SWT-4.0-130	130	5.2	11.7
Dongtai Four (H2) 300MW -South	1066	843	SWT-4.0-130	130	6.5	8.2
Fengxian - phase 1	1946	815	MySE6.45-180	180	4.5	10.8
Formosa 1 OWF - phase 2	1093	629	SWT-6.0-154	154	4.1	7.1

Wind farm	PWDS (m)	VPWDS (m)	Turbine Model	D (m)	PWD S/D	VPW DS/D
Fujian Putian City Flat Bay (Zone F)	1082	468	XE128-5MW	128	3.7	8.5
Fujian Putian City Flat Bay - 50MW	1183	860	SWT-7.0-154	154	5.6	7.7
Fujian Putian City Flat Bay Two (Zone B)	1287	730	SWT-6.0-154	154	4.7	8.4
Fuqing Xinghua Bay - phase 1 (prototype test site)	1384	739	Haliade 150-6MW	150	4.9	9.2
Guangdong Yudean Zhanjiang Wailuo -East	1478	833	MySE5.5-155	155	5.4	9.5
Guangdong Yudean Zhanjiang Wailuo -West	1459	710	MySE5.5-155	155	4.6	9.4
Huaneng Dafeng - phase 1 -North	1381	927	EN-136/4.2	136	6.8	10.2
Huaneng Rudong 300MW - South - East	1169	850	SWT-4.0-130	130	6.5	9
Huaneng Rudong 300MW - South - West	1653	831	SWT-4.0-130	130	6.4	12.7
Jiangsu Rudong Jiangjiasha H2 300MW -East	1261	819	EN-148/4.5	148	5.5	8.5
Longyuan Jiangsu Dafeng (H12) 200MW (Concession) -East	1165	569	GW 109/2500	109	5.2	10.7
Longyuan Jiangsu Dafeng (H12) 200MW (Concession) -West	1044	597	GW 109/2500	109	5.5	9.6
Longyuan Jiangsu Dafeng (H7) 200MW -North	1192	621	GW130-2.5MW	140	4.4	8.5
Longyuan Jiangsu Dafeng (H7) 200MW -South	1188	565	GW130-2.5MW	140	4	8.5
Longyuan Putian Nanri Island I - 400MW Project - phase 1	1002	579	SWT-4.0-130	130	4.5	7.7
Qidong H1	1957	539	SWT-6.25-172	172	3.1	11.4
Rudong H13	1444	770	H171-5.0MW	171	4.5	8.4
Rudong H5	1389	605	SWT-4.0-146	146	4.1	9.5
Rudong H6	1828	447	SWT-4.0-146	146	3.1	12.5
Rudong H8	1382	788	H171-5.0MW	171	4.6	8.1
Rudong Offshore Wind Farm Demonstration Project - Expansion Project (200MW) -East	1105	966	SWT-4.0-130	130	7.4	8.5
Rudong Offshore Wind Farm Demonstration Project - Expansion Project (200MW) -West	1050	1035	SWT-4.0-130	130	8	8.1

Wind farm	PWDS (m)	VPWDS (m)	Turbine Model	D (m)	PWD S/D	VPW DS/D
SPIC Binhai North H2 400MW -North	1545	726	SWT-4.0-130	130	5.6	11.9
SPIC Binhai North H2 400MW -South	1673	727	SWT-4.0-130	130	5.6	12.9
SPIC Binhai South H3 # 300MW	1355	688	SWT-4.0-146	146	4.7	9.3
SPIC Jiangsu Dafeng H3 300MW - North	1467	931	SWT-4.0-146	146	6.4	10
SPIC Jiangsu Dafeng H3 300MW - South	1475	932	SWT-4.0-146	146	6.4	10.1
SPIC Rudong H4	1734	621	SWT-4.0-146	146	4.3	11.9
SPIC Rudong H7	1046	595	SWT-4.0-146	146	4.1	7.2
Shengsi 2	1588	783	MySE6.45-180	180	4.3	8.8
Shengsi 5 + 6 -South	1612	551	SWT-6.25-172	172	3.2	9.4
Sheyang H1	2028	538	EN-148/4.5	148	3.6	13.7
Sheyang H2	1598	805	EN-148/4.5	148	5.4	10.8
Shicheng Fishing Port	1121	511	SWT-7.0-154	154	3.3	7.3
Three Gorges New Energy Jiangsu Dafeng 300MW -North	1776	727	GW140-3.3MW	140	5.2	12.7
Three Gorges New Energy Jiangsu Dafeng 300MW -South	1207	717	GW140-3.3MW	140	5.1	8.6
Xiangshui Demonstration	1296	748	SWT-4.0-130	130	5.8	10
Xinliao	2038	679	MySE6.45-180	180	3.8	11.3
Zhanjiang Xuwen-North	2133	553	MySE6.45-180	180	3.1	11.9
Zhejiang Jiaxing 1	2094	445	XE148-4000	148	3	14.1
Zhejiang Jiaxing 2	1765	755	SWT-6.0-154	154	4.9	11.5
Zhugensha H1 - Dongtai V	1057	830	G4-146	146	5.7	7.2
Zhugensha H2 -East	949	468	SWT-4.0-146	146	3.2	6.5
**Average**	**1496**	**717**		**150**	**4.9**	**10**

D: wind Turbine rotor diameter, PWDS: Prevailing wind direction spacing, VPWDS: Vertical prevailing wind direction spacing

Table S2. Available area site selection criteria.

Constraint	Bottom fixed	Floating	Data set
wind speed	> 5.0 (m/s)	> 5.0 (m/s)	Bias-corrected CMIP6 global dataset [18], MERRA-2 [19]
significant wave height	< 2.0 (m)	< 2.0 (m)	FIO-ESM v2.0 CMIP6 experiments [20]
water depth	< 60 (m)	60 ~ 1000 (m)	SRTM15+ [21]
distance from shore	> 10 (km)	-	Calculated
protect area	Exclusion	Exclusion	WDPA [22]

Table S3. Comparative overview of offshore wind potential assessments in China.

	Davidson MR [23]	Sherman P [24]	Yang Wang [25]	Xi Deng [26]	Kelly Eurek [27]	This Study
Usable Area （M km2）	-	0.4407*	0.477*	-	0.728*	0.4396-0.6423
Wind Speed Data	MERRA2	MERRA2	NCC-CMA	CCMP	NCAR	MERRA2/CMIP
Turbine Spacing	9D*9D	7D*7D	10D*5D	7D*7D	10D*5D	10D*5D
Total Number of Turbines（k）	-	423.65*	321.47*	-	1040.00*	69.264-294.4
Turbine Rating (MW)	5	8	6/6.7/8	10	3.5	4/8/11
Capacity density （MW/km^2）	5	7.69*	4.726*	9.0909*	5	1.2153-2.6186
Wake effect methods	0	5%	0	-	10%	Gaussian, Cumulative curl, Jensen
Capacity potential (TW)	-	3.39	2.254	-	3.64	0.7619-1.4032

Note: Values marked with * are computed based on the data provided in the respective papers.

The table highlights the following points:

- Usable Area: Our study calculates a range of usable areas, while some previous studies did not provide this data. Our calculations also take into account the differences between floating and bottom-fixed wind farms, providing more realistic estimates of usable area.
- Wake Effects: In our study, we explicitly model wake losses using three methods (Gaussian, Cumulative Curl, and Jensen). Many previous studies either ignored wake losses or applied simplified coefficients.
- Turbine Spacing: Our study adopted more realistic turbine spacing based on remote sensing data and actual wind farm configurations.
- Capacity Potential: The capacity potential in our study is notably lower, primarily due to the careful inclusion of wake effects and the reduced usable area. Although our potential is lower than some previous studies, we believe it provides more realistic and actionable insights.

We provide explanations for the less common datasets used:

- CCMP (ASA's Cross-Calibrated Multi-Platform Wind Vector Data): This dataset is used for estimating offshore wind resources. It is version 2.0 and is based on cross-calibration from multiple platforms, providing global wind speed data.
- NCC-CMA (National Climate Center of the China Meteorological Administration): This dataset, developed by the National Climate Center of China Meteorological Administration, has a high resolution of 3 km × 3 km and provides hourly wind data covering the period from 1980 to 2020.
- NCAR (National Center for Atmospheric Research): This dataset is used for NREL's global wind resource supply curves. It is based on a 21-year climate reanalysis dataset (1985-2005) with high temporal and spatial resolution.

Table S4. Main scenario settings for farm-scale assessment.

Scenario label	Wind speed data	Turbine size	Wind farm scale	Row number	Wake effect method	Technical potential (TWh yr^{-1})
M-8S3G	MERRA-2	8MW	Small	3	Gaussian	2648
M-8S4G	MERRA-2	8MW	Small	4	Gaussian	2617
M-8M3G	MERRA-2	8MW	Medium	3	Gaussian	3302
M-8M4G	MERRA-2	8MW	Medium	4	Gaussian	3562
M-8L3G	MERRA-2	8MW	Large	3	Gaussian	3339
M-8L4G	MERRA-2	8MW	Large	4	Gaussian	3738
S2-8S3G	SSP2-4.5	8MW	Small	3	Gaussian	2873
S2-8S4G	SSP2-4.5	8MW	Small	4	Gaussian	2840
S2-8M3G	SSP2-4.5	8MW	Medium	3	Gaussian	3589
S2-8M4G	SSP2-4.5	8MW	Medium	4	Gaussian	3869
S2-8L3G	SSP2-4.5	8MW	Large	3	Gaussian	3654
S2-8L4G	SSP2-4.5	8MW	Large	4	Gaussian	4124
S5-8S3G	SSP5-8.5	8MW	Small	3	Gaussian	2914
S5-8S4G	SSP5-8.5	8MW	Small	4	Gaussian	2873
S5-8M3G	SSP5-8.5	8MW	Medium	3	Gaussian	3663
S5-8M4G	SSP5-8.5	8MW	Medium	4	Gaussian	3921
S5-8L3G	SSP5-8.5	8MW	Large	3	Gaussian	3706
S5-8L4G	SSP5-8.5	8MW	Large	4	Gaussian	4196
M-8S3C	MERRA-2	8MW	Small	3	Cumulative Curl	2528
M-8S4C	MERRA-2	8MW	Small	4	Cumulative Curl	2457
M-8M3C	MERRA-2	8MW	Medium	3	Cumulative Curl	3124
M-8M4C	MERRA-2	8MW	Medium	4	Cumulative Curl	3297
M-8L3C	MERRA-2	8MW	Large	3	Cumulative Curl	3150
M-8L4C	MERRA-2	8MW	Large	4	Cumulative Curl	3441
M-8S3J	MERRA-2	8MW	Small	3	Jensen	2680
M-8S4J	MERRA-2	8MW	Small	4	Jensen	2644
M-8M3J	MERRA-2	8MW	Medium	3	Jensen	3322
M-8M4J	MERRA-2	8MW	Medium	4	Jensen	3567
M-8L3J	MERRA-2	8MW	Large	3	Jensen	3353
M-8L4J	MERRA-2	8MW	Large	4	Jensen	3733
M-4S3G	MERRA-2	4MW	Small	3	Gaussian	2513
M-4S4G	MERRA-2	4MW	Small	4	Gaussian	2471
M-4M3G	MERRA-2	4MW	Medium	3	Gaussian	3181
M-4M4G	MERRA-2	4MW	Medium	4	Gaussian	3406
M-4L3G	MERRA-2	4MW	Large	3	Gaussian	3248
M-4L4G	MERRA-2	4MW	Large	4	Gaussian	3630
M-11S3G	MERRA-2	11MW	Small	3	Gaussian	2733
M-11S4G	MERRA-2	11MW	Small	4	Gaussian	2715
M-11M3G	MERRA-2	11MW	Medium	3	Gaussian	3313
M-11M4G	MERRA-2	11MW	Medium	4	Gaussian	3646
M-11L3G	MERRA-2	11MW	Large	3	Gaussian	3263
M-11L4G	MERRA-2	11MW	Large	4	Gaussian	3761

Table S5. The arrangement of wind farm setting.

	Number of turbines	Rated power (MW)	Rotor diameter (m)	Farm width (m)	Farm length (m)
Three rows	24	4	130	7930	9880
		8	164	10004	12464
		11	208	12688	15808
	60	4	130	7930	17680
		8	164	10004	22304
		11	208	12688	28288
	99	4	130	7930	26130
		8	164	10004	32964
		11	208	12688	41808
Four rows	24	4	130	9230	8580
		8	164	11644	10824
		11	208	14768	13728
	60	4	130	9230	14430
		8	164	11644	18204
		11	208	14768	23088
	100	4	130	9230	20930
		8	164	11644	26404
		11	208	14768	33488

Table S6. Technical details of the wind turbine.

Wind turbine type	Rated power (MW)	Cut-in speed (m/s)	Rated wind speed (m/s)	Cut-out speed (m/s)	Rotor diameter (m)
SWT_4_130	4	4	13	25	130
V164_8	8	5	12	25	164
EW_11_208	11	3	10	25	208

Table S7. Array cable lengths by layout type and turbine model

Layout Type	Turbine Model	Internal Cables (km)	Main Cables (km)	Total Cables (km)
3-row Small, 8 columns	SWT_4_130	13.65	4.28	17.93
3-row Small, 20 columns	SWT_4_130	37.05	4.28	41.33
3-row Small, 33 columns	SWT_4_130	62.4	4.28	66.68
4-row Small, 6 columns	SWT_4_130	13	6.77	19.77
4-row Small, 15 columns	SWT_4_130	36.4	6.77	43.17
4-row Small, 25 columns	SWT_4_130	62.4	6.77	69.17
3-row Medium, 8 columns	V164_8	17.22	4.84	22.06
3-row Medium, 20 columns	V164_8	46.74	4.84	51.58
3-row Medium, 33 columns	V164_8	78.72	4.84	83.56
4-row Medium, 6 columns	V164_8	16.4	7.9	24.3
4-row Medium, 15 columns	V164_8	45.92	7.9	53.82
4-row Medium, 25 columns	V164_8	78.72	7.9	86.62
3-row Large, 8 columns	EW_11_208	21.84	5.62	27.46
3-row Large, 20 columns	EW_11_208	59.28	5.62	64.9
3-row Large, 33 columns	EW_11_208	99.84	5.62	105.46
4-row Large, 6 columns	EW_11_208	20.8	9.44	30.24
4-row Large, 15 columns	EW_11_208	58.24	9.44	67.68
4-row Large, 25 columns	EW_11_208	99.84	9.44	109.28

References

1. Hoeser, T., Feuerstein, S. & Kuenzer, C. DeepOWT: a global offshore wind turbine data set derived with deep learning from Sentinel-1 data. *Earth Syst. Sci. Data* **14**, 4251–4270 (2022).
2. 4C Offshore. Offshore Wind Farms Interactive Map. *4C Offshore* https://map.4coffshore.com/offshorewind/ (2023).
3. Technical University of Denmark (DTU) and World Bank Group. Global Wind Atlas. https://globalwindatlas.info/zh/ (2023).
4. IRENA. *Renewable Power Generation Costs in 2022*. (2023).
5. Niayifar, A. & Porté-Agel, F. Analytical Modeling of Wind Farms: A New Approach for Power Prediction. *Energies* **9**, 741 (2016).
6. Bastankhah, M. & Porté-Agel, F. Experimental and theoretical study of wind turbine wakes in yawed conditions. *J. Fluid Mech.* **806**, 506–541 (2016).
7. Jensen, N. A note on wind generator interaction. in (1983).
8. Bastankhah, M., Welch, B. L., Martínez-Tossas, L. A., King, J. & Fleming, P. Analytical solution for the cumulative wake of wind turbines in wind farms. *J. Fluid Mech.* **911**, A53 (2021).
9. Bay, C. J. *et al.* Addressing deep array effects and impacts to wake steering with the cumulative-curl wake model. *Wind Energy Sci.* **8**, 401–419 (2023).
10. Martinez, A. & Iglesias, G. Multi-parameter analysis and mapping of the levelised cost of energy from floating offshore wind in the Mediterranean Sea. *Energy Convers. Manag.* **243**, 114416 (2021).
11. Bosch, J., Staffell, I. & Hawkes, A. D. Global levelised cost of electricity from offshore wind. *Energy* **189**, 116357 (2019).
12. Maienza, C. *et al.* A life cycle cost model for floating offshore wind farms. *Appl. Energy* **266**, 114716 (2020).
13. Martinez, A. & Iglesias, G. Mapping of the levelised cost of energy for floating offshore wind in the European Atlantic. *Renew. Sustain. Energy Rev.* **154**, 111889 (2022).
14. Myhr, A., Bjerkseter, C., Ågotnes, A. & Nygaard, T. A. Levelised cost of energy for offshore floating wind turbines in a life cycle perspective. *Renew. Energy* **66**, 714–728 (2014).
15. Stehly, T., Duffy, P. & Hernando, D. M. 2022 Cost of Wind Energy Review. (2023) doi:10.2172/2278805.
16. Nunemaker, J., Shields, M., Hammond, R. & Duffy, P. *ORBIT: Offshore Renewables Balance-of-System and Installation Tool*. NREL/TP-5000-77081, 1660132, MainId:26027 https://www.osti.gov/servlets/purl/1660132/ (2020) doi:10.2172/1660132.
17. Bjerkseter, C. & Ågotnes, A. (2013) - Levelised Costs of Energy for Offshore Floating Wind Turbine Concepts.
18. Xu, Z., Han, Y., Tam, C.-Y., Yang, Z.-L. & Fu, C. Bias-corrected CMIP6 global dataset for dynamical downscaling of the historical and future climate (1979–2100). *Sci. Data* **8**, 293 (2021).
19. Global Modeling and Assimilation Office (GMAO) (2015), MERRA-2 inst3_3d_asm_Np: 3d,3-Hourly,Instantaneous,Pressure-Level,Assimilation,Assimilated Meteorological Fields V5.12.4, Greenbelt, MD, USA, Goddard Earth Sciences Data and Information Services Center (GES DISC), Accessed: [Data Access Date], 10.5067/QBZ6MG944HW0.
20. Qiao, F. Simulated long-term monthly ocean surface waves parameters from FIO-ESM v2.0 CMIP6 experiments for past, present, and future climate research. (2020) doi:10.6084/m9.figshare.c.4819503.v1.

21. Tozer, B. *et al.* Global Bathymetry and Topography at 15 Arc Sec: SRTM15+. *Earth Space Sci.*
22. IUCN and UNEP-WCMC. The World Database on Protected Areas (WDPA). https://data.apps.fao.org/catalog/dataset/bfcb8c96-648c-4c31-9702-20fc5d4d5b49 (2020).
23. Davidson, M. R., Zhang, D., Xiong, W., Zhang, X. & Karplus, V. J. Modelling the potential for wind energy integration on China's coal-heavy electricity grid. *Nat. Energy* **1**, 1–7 (2016).
24. Sherman, P., Chen, X. & McElroy, M. Offshore wind: An opportunity for cost-competitive decarbonization of China's energy economy. *Sci. Adv.* **6**, eaax9571 (2020).
25. Wang, Y., Chao, Q., Zhao, L. & Chang, R. Assessment of wind and photovoltaic power potential in China. *Carbon Neutrality* **1**, 15 (2022).
26. Deng, X. *et al.* Offshore wind power in China: A potential solution to electricity transformation and carbon neutrality. *Fundam. Res.* S266732582200440X (2022) doi:10.1016/j.fmre.2022.11.008.
27. Eurek, K. *et al.* An improved global wind resource estimate for integrated assessment models. *Energy Econ.* **64**, 552–567 (2017).
