# Supplementary material for: Substantially lower estimates in China’s offshore wind potential using farm-scale spatial modeling and wake effects
Source: Nat Commun. 2026 Jan 26;17:2043. doi: 10.1038/s41467-026-68655-2 (PMC12946245; doi:10.1038/s41467-026-68655-2)
Supplement: Supplementary file 2 — Transparent Peer Review file [file 41467_2026_68655_MOESM2_ESM.pdf]

nature portfolio

Peer Review File

Substantially Lower Estimates in China's Offshore Wind Potential Using Farm-Scale Spatial Modeling and Wake Effects

Corresponding Author: Professor Chuan Zhang

Parts of this Peer Review File have been redacted as indicated to remove third-party material.

This file contains all reviewer reports in order by version, followed by all author rebuttals in order by version.

Version 0:

Reviewer comments:

Reviewer #1

(Remarks to the Author)
Review of manuscript #NCOMMS-25-10524
Title: China's offshore wind power potential revisited: a realistic appraisal suggests substantially lower estimates than precedent studies

The study presents a detailed, farm-level evaluation of offshore wind power potential in China, incorporating realistic turbine layouts derived from remote sensing data, wake loss modeling, and future climate scenarios. The findings also show that China's offshore wind potential is significantly lower than previous estimates. The study is very relevant to the current understanding of wind energy potential and its projected impact on the overall energy mix. The underprediction of potential is an important realization when it comes to planning and policy activities, not just for the studied country, but also for other countries with very aggressive approach to adoption of offshore wind. The manuscript is very understandable and well written in terms of English language and style. Despite this, the overall presentation of the manuscript does not facilitate the reader in understanding the discussion and context.

Major reorganization of the manuscript sections is necessary due to the following observations:
1. The Materials and Methods section is presented at the end of the paper. This is an awkward position since the discussion refers to this section as early as line #98 of page 3. Since the section is an important part of the building up of the study storyline, it has to be put near the start so that the reader is not forced to shuffle between the first few pages and the last pages just to understand the topic being discussed. Presenting it earlier will avoid questions being raised due to incomplete setting of the stage.
2. The manuscript cross references multiple figures (S1, S2, etc.) that are part of the supplementary document. This is not appropriate since the paper needs to be intact and should stand on its own. Supplemental information may help in extended appreciation of the subject matter but may not be necessary to make the paper whole. If the authors' desire is to make the cross references intimate parts of the arguments and analysis, these figures should be integral parts of the paper, not supplemental information.
3. Most of the figures are composite images of a lot of information. It is suggested that these figures be deconstructed into more manageable illustrations of ideas as they are more understandable piecewise. Having multiple ideas illustrated and combined into one figure also causes these illustrations to have very tiny components, including the text within. They have become unreadable and difficult to understand even when maximised across the width of the paper. These should be revised so that the figures are separate.
4. The authors refer to "vertical windward spacing" multiple times. What does this mean relative to "windward spacing"? Do they mean lateral/crosswind spacing? "Vertical spacing" does not make sense without proper reference to either cardinal directions, or wind directions. A better term should be used instead of this.
5. Is the "Discussion" section (line 308, page 8) the conclusion section? It reads like that. The conclusion section should be properly labeled so that there is closure in the paper.

The authors present a cost model considering water depth and distance to shore and conclude that LCOE is significantly

affected by these factors. This is an obvious conclusion. The farther the wind farm is from the shore, the longer are the submarine cables required. The deeper the bathymetry is, the more expensive the floating structure and foundation. These factors are already incorporated in the overall cost considerations. There is really no need to highlight this. Additionally, the authors argue that higher capacity factors do not necessarily mean lower LCOE. This is also true and obvious. The LCOE already considers that. Higher capacity factors result in higher annual energy generation (numerator of the LCOE equation). However, LCOE is also greatly influenced by cost (denominator of the LCOE equation). That is the reason why LCOE is used as a metric for energy development since it considers almost all of the necessary factors that influence energy production (excluding the time value of money, where NPV is a more useful metric.)

Since the paper presents predicted LCOE, it may be prudent to compare these to the current values of LCOE in recent literature, particularly IRENA data and trends. It is necessary to place these computed LCOE against the current rates since that influences policy directions. Additionally, since the study suggests that power potential is substantially lower than previous estimates, what are the LCOEs of the previous studies and how do these compare to the current values?

(Remarks on code availability)

Reviewer #2

(Remarks to the Author)
The manuscript presents a comprehensive analysis of China’s offshore wind power potential, incorporating wake loss effects, economic feasibility, and technical constraints. The study is relevant and provides valuable insights into wind energy research.

The authors compare three wake loss models and select wind turbines based on existing wind farms, enhancing the practical relevance of their approach. They utilize MERRA-2 reanalysis data along with a bias-corrected CMIP6 dataset for dynamic downscaling. While the inclusion of bias correction improves reliability, further clarification on its methodology and effectiveness in reducing model biases would strengthen the study. Additionally, a more detailed discussion of how their findings compare with previous literature, sensitivity analyses of wake loss models, and transparency in economic assumptions would enhance credibility. While the methodology appears sound, additional details on wake modeling and economic constraints would improve reproducibility.

Overall, the study is significant, but revisions are needed to clarify validation approaches, and methodological transparency before publication.

(Remarks on code availability)
The code has been successfully executed, and I did not encounter any errors.

Reviewer #3

(Remarks to the Author)
Title: China’s offshore wind power potential revisited: a realistic appraisal sug-gests substantially lower estimates than precedent studies

In this study, the authors assess the potential of offshore wind energy in China at the plant level, considering remote sensing data, wake loss models, and future climate scenarios. The article is highly critical and interesting, but needs major changes before publication.

Introduction:

Clearly define the objectives and contribution of the work to the scientific community.

Main text:

In the wake effect analysis, the statement (lines 187-188): “Previous studies have often chosen to ignore wake losses or apply a generalized reduction factor using a fixed coefficient” requires citation. Many offshore wind studies determine the wake effect of each wind turbine individually, for example: https://doi.org/10.1016/j.jclepro.2023.139484
https://doi.org/10.1016/j.renene.2021.10.058

What about other designs, for example, staggered wind turbines?

Discussion:

The discussion is critical, but what's happening in the rest of the world? Could it serve as a reference for China? Is it possible to adopt strategies from other countries to guide their development in offshore wind energy?

(Remarks on code availability)

The materials and methods are sufficient to reproduce the article.

Reviewer #4

(Remarks to the Author)
Comments on Main Body
Lines 121–128:
The significantly lower potential obtained in your study appears to be primarily due to the reduced usable area compared to previous studies. I believe your assessment is more realistic than others, as it is impractical to assume that every ocean space can accommodate offshore wind turbines (such as in S1 and S2 from the case studies). However, making a direct comparison between your results and previous literature is challenging without a clear understanding of the assumptions in those studies. I suggest presenting a comparative table that outlines key methodological differences between your study and the literature (8, 9, 11, 17, 23). This table could include details such as usable area, wind speed data sources, turbine spacing assumptions, total number of turbines, turbine rating, capacity density (MW/km²), and wake effect methods (e.g., Gaussian, cumulative curl, Jensen, or assumed wake loss ratio). Providing this structured comparison would offer a clearer perspective on the novelty of your work, rather than focusing solely on differences in TWh/year, which are largely influenced by the reduction in usable area.
Lines 379–386:
Your rationale for selecting the number of rows and spacing between turbines based on prevailing wind directions is well-founded. I understand that the number of columns was determined using wind farm sizes from IRENA. Could you provide a visual example of Chinese offshore wind farms with a similar number of columns as those in the 3×(Large) layout cases for your 3-row and 4-row configurations? In Europe and the U.S., such large numbers of columns are uncommon, but this may be different in China. Including such a reference would help validate the realism of these layouts.
Line 428:
Please ensure consistency by using "CapEx" instead of "CapeEx" throughout the "Calculation of Costs" section.
Lines 442–447:
• Please define "n" explicitly (I assume it refers to the project design life) and specify the value used.
• Line 446: The provided formula corresponds to the capital recovery factor, not the Fixed Charge Rate (FCR). The FCR is the capital recovery factor multiplied by the project finance factor. In the latest Cost of Wind Energy Review, we assumed a project finance factor of 105% (see NREL Report). Please incorporate this factor into your calculation and state your assumption.
• Line 447: Is the Weighted Average Cost of Capital (WACC) a nominal or real value? This distinction is important, as it determines whether the FCR should be real or nominal. At NREL, we typically use the real FCR for LCOE calculations.
Line 451:
• Are array cable costs included in the analysis? If so, please clarify, as these costs vary based on layout choice.
• Does C_found include the transition piece for monopiles? Please specify.
• Does C_ins cover the installation costs for components beyond the foundation (e.g., turbines, substations, array cables, export cables)? Please clarify.
• It appears that soft CapEx elements such as construction financing, installation contingency, procurement contingency, and construction insurance are not considered. Please either include these costs or explicitly state that they are excluded from the LCOE calculations. If needed, NREL's open-source model contains relevant formulas and industry-based factors (see ORBIT Code).
Line 665:
Your LCOE values for floating and fixed-bottom technologies fall within a reasonable range, considering this is a resource assessment. However, please refer to my earlier comments on refining your assumptions in both the main body and supplemental information.
Line 676:
Did all literature sources (8, 9, 11, 17, 23) assume a 10% wake loss ratio? If different papers used varying wake loss ratios, I recommend incorporating those values into your plots. Highlighting differences in wake loss assumptions may further emphasize the novelty of your work.

Comments on Supplemental Information
Line 54:
How are the distances (D) calculated? Is this based on the closest point to shore? Do you have a predefined set of potential Points of Interconnection (POI), and is transmission distance determined using the nearest POI? Similarly, for ports, do you calculate distance to the closest port? If D represents both transmission distance and distance to port and is based solely on the nearest shoreline, please acknowledge this simplification. Additionally, clarify that installation costs are influenced by port distance, while transmission costs depend only on transmission distance.
Line 69:
The CapEx estimates derived from this formula fall within an acceptable range, at least for the U.S. offshore wind market. Could you provide a reference or background information justifying the use of this formula?
Line 81:
Does this cost estimate include the transition piece (TP), or are you assuming a TP-less monopile? The values seem reasonable, but could you provide a reference or the rationale behind this formula?
Line 83:
Similar to the monopile comment—please provide a reference for this cost estimate.
Line 85:
Floating substructure costs are nearly as high as turbine CapEx. While the estimated costs appear somewhat low, they remain within a reasonable range. Could you provide a reference or background explanation for this formula?

Lines 95–96:
Both cost estimates seem largely insensitive to transmission distance, which is unexpected. For example, AC transmission costs at 10 km and 1000 km are nearly identical ($34.5/kW vs. $34.51/kW). Could there be an issue with the formula? Please verify.
Lines 106–111:
If these values represent only the substructure installation costs, they appear reasonable. However, if they account for the entire wind farm's installation, they seem considerably low. The 2024 Cost of Wind Energy Review estimates installation costs at $830/kW for fixed-bottom and $1,058/kW for floating, assuming port distances of 116 km and 189 km, respectively. Please provide a reference or justification for these formulas.
Lines 112–117:
What is the basis for using 2.3%? In NREL's open-source model (ORBIT), this value accounts for site auction prices, site assessment plans, construction plans, and installation plans. Our assumptions can be found here: ORBIT Code. Please provide a reference or rationale for your assumption.
Lines 118–124:
Similarly, what is the basis for using 2.4%? In ORBIT, we assume 17.5% of installation CapEx (see ORBIT Code). Please provide a reference or justification.
Lines 126–136:
The cost appears to be largely insensitive to D. Could you provide a reference or explain the reasoning behind these formulas?

(Remarks on code availability)

Reviewer #5

(Remarks to the Author)
I co-reviewed this manuscript with one of the reviewers who provided the listed reports. This is part of the Nature Communications initiative to facilitate training in peer review and to provide appropriate recognition for Early Career Researchers who co-review manuscripts.

(Remarks on code availability)

Version 1:

Reviewer comments:

Reviewer #1

(Remarks to the Author)
The authors have addressed all concerns raised by the reviewer. Though they did not agree with all of the comments and have not adopted all suggested actions, they have articulated the rationale behind their decision to retain some of the components of the manuscript as presented and only made improvements that are within the constraints of the journal guidelines. The revisions already have significantly improved the manuscript.

(Remarks on code availability)

Reviewer #2

(Remarks to the Author)
The authors have carefully addressed the earlier comments and revised the manuscript accordingly. They have clearly acknowledged the limitations of their work and provided appropriate directions for future research. The revisions have strengthened the clarity and scientific contribution of the study. I find the manuscript to be suitable for publication in its current form.

(Remarks on code availability)
I was able to run the code successfully with the provided dataset, and it reproduces the results reported in the manuscript. However, for broader applicability, the code requires additional preprocessing steps to handle other datasets. In particular, while climate and wind data are commonly distributed in NetCDF format, the current implementation is limited to CSV input. This restricts direct usability for researchers working with large-scale datasets. I recommend that the authors extend the code to support NetCDF input in future updates, as this would substantially improve flexibility and community adoption. Furthermore, enhancing the README with step-by-step instructions on preprocessing, input formatting, and example workflows would strengthen reproducibility and make the code a more practical resource for the wider research community.

Reviewer #3

(Remarks to the Author)

Dear authors:
Thank you for the modifications made; the work has improved in scientific quality.

(Remarks on code availability)

Reviewer #4

(Remarks to the Author)
This paper makes a valuable and timely contribution to the literature on renewable energy resource assessments. By integrating high-resolution remote sensing data, realistic turbine layouts, multiple wake modeling approaches, and future climate scenarios, the study provides one of the most comprehensive and realistic farm-level evaluations of China's offshore wind potential to date. The finding that potential is considerably lower than earlier estimates—primarily due to reduced usable area and more accurate wake loss modeling—represents an important correction that will help policymakers and planners set more achievable energy targets.

I particularly commend the authors for the careful attention they paid in addressing reviewer feedback. The inclusion of a structured comparative table detailing methodological differences with prior studies significantly enhances transparency and highlights the novelty of their approach. Similarly, the addition of real-world turbine layout examples from Chinese offshore wind farms, along with open access shapefiles, greatly strengthens the credibility and reproducibility of the results. The revisions to cost modeling, including the explicit treatment of soft CapEx elements, array cable costs, and financial assumptions (WACC, FCR), bring the economic analysis in line with best practices in the field and ensure robustness. Clarifying assumptions on distances, foundation costs, and substructure cost projections further adds to the methodological rigor.

The study's results—showing lower overall potential but more realistic assessments of technical and economic feasibility, particularly for floating wind in deep-water regions—will be of high value for both the research community and energy policymakers. By carefully distinguishing between bottom-fixed and floating offshore wind, the authors provide actionable insights into the trade-offs between technical potential, spatial constraints, and costs.

Overall, this is a well-revised manuscript that makes a meaningful and original contribution to offshore wind energy research, and I support its publication.

(Remarks on code availability)

Reviewer #5

(Remarks to the Author)
I co-reviewed this manuscript with one of the reviewers who provided the listed reports. This is part of the Nature Communications initiative to facilitate training in peer review and to provide appropriate recognition for Early Career Researchers who co-review manuscripts.

(Remarks on code availability)

Open Access This Peer Review File is licensed under a Creative Commons Attribution 4.0 International License, which permits use, sharing, adaptation, distribution and reproduction in any medium or format, as long as you give appropriate credit to the original author(s) and the source, provide a link to the Creative Commons license, and indicate if changes were made.
In cases where reviewers are anonymous, credit should be given to 'Anonymous Referee' and the source.
The images or other third party material in this Peer Review File are included in the article's Creative Commons license, unless indicated otherwise in a credit line to the material. If material is not included in the article's Creative Commons license and your intended use is not permitted by statutory regulation or exceeds the permitted use, you will need to obtain permission directly from the copyright holder.
To view a copy of this license, visit https://creativecommons.org/licenses/by/4.0/

REVIEWER COMMENTS

We sincerely thank the reviewers and editors for their thoughtful and constructive comments, which have greatly helped us improve the manuscript.

Reviewer #1 (Remarks to the Author):

Title: China's offshore wind power potential revisited: a realistic appraisal suggests substantially lower estimates than precedent studies

The study presents a detailed, farm-level evaluation of offshore wind power potential in China, incorporating realistic turbine layouts derived from remote sensing data, wake loss modeling, and future climate scenarios. The findings also show that China's offshore wind potential is significantly lower than previous estimates. The study is very relevant to the current understanding of wind energy potential and its projected impact on the overall energy mix. The underprediction of potential is an important realization when it comes to planning and policy activities, not just for the studied country, but also for other countries with very aggressive approach to adoption of offshore wind. The manuscript is very understandable and well written in terms of English language and style. Despite this, the overall presentation of the manuscript does not facilitate the reader in understanding the discussion and context.

Major reorganization of the manuscript sections is necessary due to the following observations:
1. The Materials and Methods section is presented at the end of the paper. This is an awkward position since the discussion refers to this section as early as line #98 of page 3. Since the section is an important part of the building up of the study storyline, it has to be put near the start so that the reader is not forced to shuffle between the first few pages and the last pages just to understand the topic being discussed. Presenting it earlier will avoid questions being raised due to incomplete setting of the stage.

We appreciate your feedback regarding the placement of the Materials and Methods section. We would like to clarify that the current placement at the end of the manuscript follows the formatting guidelines set by Nature Communications submission page , detailed in their official submission guide PDF, and Nature Communications formatting instructions, *which typically require to place these sections after the main text.*

Within such constrain, to help the reader follow the storyline more easily, we have ensured that all key methods and data sources are briefly summarized or referenced in the main text, then with full methodological details documented in the Materials and Methods section. We hope

this approach maintains clarity while also adhering to the journal's required structure.

2. The manuscript cross references multiple figures (S1, S2, etc.) that are part of the supplementary document. This is not appropriate since the paper needs to be intact and should stand on its own. Supplemental information may help in extended appreciation of the subject matter but may not be necessary to make the paper whole. If the authors' desire is to make the cross references intimate parts of the arguments and analysis, these figures should be integral parts of the paper, not supplemental information.

We fully appreciate the reviewer's concern. In the revised manuscript, we have carefully ensured that the manuscript remains self-contained and scientifically coherent on its own. We have minimized cross-reference between the main manuscript and supplemental information wherever possible as the reviewer suggest. Yet it is also acknowledged that referencing figures in the Supplementary Information seems unavoidable in several cases due to the limitation of figure numbers. In such cases, we have made sure that the formatting is consistent with the editorial policy of Nature Communications, which encourages placing technical, methodological, or supporting content in the Supplementary section to keep the main text focused and concise. The references to Supplementary Figures (e.g., Fig. S1 and S2) serve to provide additional visual detail and expanded technical insights for interested readers, rather than conveying any essential argument that would compromise the understanding of the main text. If the editors or reviewers believe that specific Supplementary Figures are critical to the core narrative, we are happy to move them into the main manuscript as requested.

3. Most of the figures are composite images of a lot of information. It is suggested that these figures be deconstructed into more manageable illustrations of ideas as they are more understandable piecewise. Having multiple ideas illustrated and combined into one figure also causes these illustrations to have very tiny components, including the text within. They have become unreadable and difficult to understand even when maximised across the width of the paper. These should be revised so that the figures are separate.

We thank the reviewer for this helpful suggestion. The idea of combining multiple panels into composite figures was to facilitate holistic comparison between closely related variables and scenarios. However, we fully acknowledge the reviewer's concern that this approach may result in overly dense figures with small, hard-to-read text elements.

In response, we have taken the following actions:

- *Increased the font size and resolution of all figures to improve readability;*

- *Restructured Fig. 3 by separating the original multi-panel layouts into two standalone figures, each focusing on a distinct analytical aspect;*
- *Updated the figure captions accordingly for clarity.*

We believe these revisions will significantly enhance the interpretability of our figures, should the editors or reviewers recommend further deconstruction of other figures, we are happy to make additional refinements.

4. The authors refer to "vertical windward spacing" multiple times. What does this mean relative to "windward spacing"? Do they mean lateral/crosswind spacing? "Vertical spacing" does not make sense without proper reference to either cardinal directions, or wind directions. A better term should be used instead of this.

Thank you for your feedback. Indeed, the term " vertical windward spacing" in our manuscript refers to crosswind spacing. We have revised the manuscript to replace all instances of " vertical windward spacing" with "crosswind spacing" to enhance accuracy.

5. Is the "Discussion" section (line 308, page 8) the conclusion section? It reads like that. The conclusion section should be properly labeled so that there is closure in the paper.

Thank you for bringing this to our attention. As far as our understanding of the journal's formatting requirements, it requires only a "Discussion" section and not a separate "Conclusion", in such case we may need to retain the "Discussion" section as it is. However, we have clearly summarized the main findings and their significance in this section, ensuring that the "Discussion" appropriately serves the dual purpose of interpretation and closure.

6. The authors present a cost model considering water depth and distance to shore and conclude that LCOE is significantly affected by these factors. This is an obvious conclusion. The farther the wind farm is from the shore, the longer are the submarine cables required. The deeper the bathymetry is, the more expensive the floating structure and foundation. These factors are already incorporated in the overall cost considerations. There is really no need to highlight this. Additionally, the authors argue that higher capacity factors do not necessarily mean lower LCOE. This is also true and obvious. The LCOE already considers that. Higher capacity factors result in higher annual energy generation (numerator of the LCOE equation). However, LCOE is also greatly influenced by cost (denominator of the LCOE equation). That is the reason why LCOE is used as a metric for energy development since it considers almost all of the necessary factors that influence energy production (excluding the time value of money, where NPV is a more useful metric.)

We sincerely appreciate the reviewer's careful evaluation and feedback on our work. We fully understand the reviewer's observation that "the impact of water depth and distance to shore on LCOE" and "higher capacity factors do not necessarily correspond to lower LCOE" are well-established concepts. However, we would like to further clarify the substantive contributions of this study and explain why these seemingly "obvious" factors still present significant potential for misjudgment in practical assessment and policy related decision-making.

1. Comprehensive empirical analysis of depth and distance effects on LCOE. While water depth and distance to shore are indeed recognized variables in LCOE models, our contribution lies in systematically quantifying their impacts through high-resolution geographic observational data covering the entire national territory, based on a detailed farm-level modeling. We developed and validated a comprehensive LCOE cost model that distinguishes between bottom-fixed and floating wind turbines while incorporating multiple climate scenarios and wind speed data (see Fig. 4A-D and SI Fig. S7-S9). These analyses reveal a critical nonlinear characteristic: increased offshore distance enhances wind speeds but simultaneously increases water depth, leading to rapidly escalating installation, operation and maintenance, and transmission costs. Different regions (such as Fujian versus Guangdong) show significantly different LCOE values under similar capacity factors, precisely due to variations in geographic-economic parameters (detailed comparison in Fig. 4C and Fig. S6 overlay analysis). Therefore, we argue that the "apparent existence" of these cost factors is not equal to "adequate quantification," thus we address this gap by providing nationwide cost mapping based on real-world remote sensing and GIS data, offering important benchmark for regional potential assessment and cost quantification.

2. Systematic validation of the non-monotonic relationship between capacity factor and LCOE. We fully agree with the reviewer that LCOE inherently considers capacity factor effects already. However, our work provides the first farm-level empirical evidence across thousands of wind farms throughout China, clearly demonstrating the geographic distribution patterns of this non-monotonic relationship. For instance, in the comparative analysis of Fig. S6 and Fig. 4, we found that in Zhejiang and Fujian provinces, higher capacity factors indeed result in lower LCOE, while in Guangdong and Shandong provinces, despite high wind speeds and capacity factors, LCOE remains elevated due to greater water depths and distances from shore. Again, such "geographic counterexamples" have practical implications for real-world energy planning and investment decision making, indicating that capacity factor or wind speed alone cannot serve as preliminary screening criteria and must be analyzed in conjunction with LCOE spatial heterogeneity.

In conclusion, while the theoretical relationships between these factors and LCOE might have been qualitatively established, our study further bridges the knowledge gap between conceptual

understanding and numeric quantification by providing the first comprehensive, spatially explicit assessment of offshore wind economics across China, which we believe feature an important advancement of our study.

7. Since the paper presents predicted LCOE, it may be prudent to compare these to the current values of LCOE in recent literature, particularly IRENA data and trends. It is necessary to place these computed LCOE against the current rates since that influences policy directions. Additionally, since the study suggests that power potential is substantially lower than previous estimates, what are the LCOEs of the previous studies and how do these compare to the current values?

We thank the reviewer for this valuable comment regarding the needs to contextualize our computed LCOE values relative to current literature, including those authoritative sources such as IRENA and prior offshore wind potential assessments. Firstly, we can clarify that the LCOE values presented in our study are not future projections but are based on 2022 cost assumptions and technical parameters, enabling direct comparison with internationally reported values for the same year. As suggested, we have conducted a systematic comparison between our modeled LCOE results and representative scenarios reported in authoritative literature and institutional datasets. It turns out that:

For bottom-fixed offshore wind, our LCOE estimates range from 48.79 to 158.65 USD/MWh, with a mean of 106.89 USD/MWh and a median of 108.23 USD/MWh. These values align well with the IRENA-reported[1] global average of 81 USD/MWh for 2022 and the NREL reference value of 95 USD/MWh for a representative U.S. fixed-bottom site[2].

For floating offshore wind, our modeled LCOEs span 82.44 to 241.62 USD/MWh, with a mean of 138.98 USD/MWh and a median of 137.45 USD/MWh, which are comparable to NREL's 2022 estimate of 145 USD/MWh for a U.S. West Coast floating project[2].

Additionally, Peter Sherman et al. [3] report an LCOE range of 20–140 USD/MWh depending on scenario assumptions, while Bosch et al. [4] estimate capital costs of 70–300 USD/kW—which, when converted to LCOE using standard cost models, support a similar magnitude to our results. Such consistency across sources reinforces the realism of our cost assumptions and modeling approach.

While the overall cost magnitude is comparable, our study exhibits a notable distinguishing feature. We explicitly capture the effects of wind farm scale on LCOE through high-resolution spatial modeling across multiple layout sizes (small, medium, large). The results show that larger wind farms generally benefit from economies of scale, as reflected in systematically lower LCOEs within our simulations—especially under similar geospatial and climate conditions. This scale effect emerges due to the more efficient distribution of fixed costs (e.g.,

substation, export cable) and improved utilization of transmission infrastructure across larger installed capacities. Such results suggest that project aggregation or centralized offshore wind planning could help lower costs in practice. The related implications are now explicitly discussed in the revised manuscript.

Finally, as suggested by Reviewer #4, we have added a comparative table (see Table 2) that outlines the key methodological differences between our study and previous assessments, including assumptions on usable area, turbine spacing, wake loss ratios, and wind resource datasets. This provides further context for why earlier studies may have reported higher technical potential and lower LCOE and emphasizes the realism and practical relevance of our spatially explicit cost modeling. We hope these additions could significantly strengthen the novelty of our manuscript and address the reviewer's concern about situating our LCOE findings within the broader literature.

Reviewer #2 (Remarks to the Author):

The manuscript presents a comprehensive analysis of China's offshore wind power potential, incorporating wake loss effects, economic feasibility, and technical constraints. The study is relevant and provides valuable insights into wind energy research.

The authors compare three wake loss models and select wind turbines based on existing wind farms, enhancing the practical relevance of their approach. They utilize MERRA-2 reanalysis data along with a bias-corrected CMIP6 dataset for dynamic downscaling. While the inclusion of bias correction improves reliability, further clarification on its methodology and effectiveness in reducing model biases would strengthen the study. Additionally, a more detailed discussion of how their findings compare with previous literature, sensitivity analyses of wake loss models, and transparency in economic assumptions would enhance credibility. While the methodology appears sound, additional details on wake modeling and economic constraints would improve reproducibility.

Overall, the study is significant, but revisions are needed to clarify validation approaches, and methodological transparency before publication.

Reviewer Comment 1:

"While the inclusion of bias correction improves reliability, further clarification on its methodology and effectiveness in reducing model biases would strengthen the study."

We thank the reviewer for raising this important point. The bias-corrected CMIP6 dataset we employed was developed by Xu et al.[5], using the MVT (Mean-Variance-Trend) correction method. This method improves the reliability of dynamical downscaling by correcting not only the climatological mean and interannual variance of wind speed data, but also the long-term non-linear trend using the ensemble empirical mode decomposition (EEMD) from 18 CMIP6 models.

Specifically, the historical (1979–2014) and future (2015–2100, SSP245 and SSP585) climate data were corrected such that the corrected series aligns with ERA5 in terms of mean and variability, while preserving the non-linear future trend projected by the CMIP6 ensemble mean. This approach reduces systematic biases that would otherwise be inherited by the downscaling model.

To demonstrate the effectiveness of the bias correction, Xu et al. showed that the corrected GCM data eliminate mean and variance biases and better match the distribution and variability of ERA5 wind and temperature fields (see Fig. 2–4 in their paper). We have cited this source

in the revised Methods section and added a brief explanation summarizing the procedure and its role in improving wind resource assessment.

Reviewer Comment 2:

"A more detailed discussion of how their findings compare with previous literature..."

Thank you for the suggestion. In the revised Discussion section, we now provide a clearer comparison between our results and serval key previous assessments of China's offshore wind potential. See the table below for details. For a full explanation of the assumptions and parameter settings used in this comparison, please also refer to our response to Reviewer #4, Comment 1 on page 24 of the letter.

Table 1. Comparative Overview of Offshore Wind Potential Assessments in China

	Davidson MR [6]	Sherman P [3]	Yang Wang [7]	Xi Deng [8]	Kelly Eurek [9]	This Study
Usable Area (M km2)	-	0.4407*	0.477*	-	0.728*	0.4396-0.6423
Wind Speed Data	MERRA2	MERRA2	NCC-CMA	CCMP	NCAR	MERRA2/CMIP
Turbine Spacing	9D*9D	7D*7D	10D*5D	7D*7D	10D*5D	10D*5D
Total Number of Turbines (k)	-	423.65*	321.47*	-	1040.00*	69.264-294.4
Turbine Rating (MW)	5	8	6/6.7/8	10	3.5	4/8/11
Capacity density (MW/km^2)	5	7.69*	4.726*	9.0909*	5	1.2153-2.6186
Wake effect methods	0	5%	0	-	10%	Gaussian, Cumulative curl, Jensen
Capacity potential (TW)	-	3.39	2.254	-	3.64	0.7619-1.4032

Reviewer Comment 3:

"Sensitivity analyses of wake loss models..."

We thank the reviewer for emphasizing the importance of sensitivity analysis. In our study, we conducted a systematic comparison across three widely used wake loss models: the Gaussian velocity deficit model, the classic Jensen (Park) model, and the Cumulative Curl model.

The results of this sensitivity analysis are presented in Figure 5, which illustrates both national-scale power potential and LCOE variations under different combinations of wake models, turbine types, and climate datasets. We find that:

- *The Gaussian and Jensen models yield broadly similar estimates of power generation potential and LCOE under the same conditions.*
- *The Cumulative Curl model, which simulates more complex wake interactions and recovery, consistently predicts 10–15% lower technical potential and higher LCOE values than the other two models, especially in densely packed wind farm configurations.*

These differences are clearly shown in Figure 5B and 5C, where power potential and LCOE curves diverge by wake model under the same wind speed forcing and turbine capacity. The analysis demonstrates that the choice of wake loss model can significantly influence national potential assessments and cost metrics. We have added corresponding statements in the revised Results and Discussion sections to emphasize these findings.

"Additionally, the study quantified wake losses, which were found to be considerably higher than the typically cited 10%, indicating the needs for more sophisticated wake loss models. This finding underscores the complexity of accurately predicting energy production from wind farms, as wake effects vary significantly depending on factors like turbine placement and atmospheric conditions. In addition, the spacing standards of wind turbines, the arrangement of wind farms, etc. will greatly affect the wake losses and overall potential, highlighting the importance of effective planning before construction."

Reviewer Comment 4:

"Transparency in economic assumptions would enhance credibility."

A: We appreciate this recommendation. We have revised the Methods and Supplementary Information to make sure all economic parameters used in the LCOE calculation as transparent. This includes:

- *Turbine CAPEX and OPEX values (by type)*

- *Fixed charge rate (6.5%), project lifetime (25 years)*
- *Bathymetry and cable length cost coefficients*
- *No inclusion of subsidies or carbon price assumptions*

A full list of parameter values is now included in Table S6. We also compare these assumptions with recent global LCOE values from IRENA and NREL in the revised Discussion and SI.

Reviewer #2 (Remarks on code availability):

The code has been successfully executed, and I did not encounter any errors.

We are glad that the code works!

Reviewer #3 (Remarks to the Author):

Title: China's offshore wind power potential revisited: a realistic appraisal sug-gests substantially lower estimates than precedent studies

In this study, the authors assess the potential of offshore wind energy in China at the plant level, considering remote sensing data, wake loss models, and future climate scenarios. The article is highly critical and interesting, but needs major changes before publication.

Introduction:

Clearly define the objectives and contribution of the work to the scientific community.

We thank the reviewer for this helpful suggestion. In response, we have revised the final paragraph of the Introduction to more clearly articulate the objectives and contributions of our study. Specifically, we now emphasize that:

a) We provide the first high-resolution, farm-scale assessment of China's offshore wind power potential that integrates realistic turbine layouts and spacing patterns derived from remote sensing data;

b) We incorporate multiple wake loss models—including Gaussian, Jensen, and Cumulative Curl formulations—to quantify and compare wake effects under different turbine arrangements and climate scenarios;

c) We use a bias-corrected CMIP6 dataset to evaluate both present-day and future wind resources under SSP climate pathways;

d) We estimate spatially resolved technical potential and levelized cost of electricity (LCOE), offering planning-relevant insights grounded in realistic spatial and economic constraints.

These clarifications have been included in the revised Introduction (page 2), to better communicate the scope and scientific contributions of our work.

Page2:

Objectives: *"This study aims to provide a realistic and spatially explicit assessment of China's offshore wind power potential under both current and future climate conditions. To achieve this, we integrate wind farm-scale turbine layout statistics derived from remote sensing data, multiple wake loss models, and a bias-corrected CMIP6 climate dataset."*

Contribution: *"On the other hand, this work introduces a new framework for wind farm-scale offshore resource assessment, offering scientific insights into the spatial, technical, and*

economic dynamics of wind energy deployment."

Main text:

In the wake effect analysis, the statement (lines 187-188): "Previous studies have often chosen to ignore wake losses or apply a generalized reduction factor using a fixed coefficient" requires citation. Many offshore wind studies determine the wake effect of each wind turbine individually, for example: https://doi.org/10.1016/j.jclepro.2023.139484 https://doi.org/10.1016/j.renene.2021.10.058

We thank the reviewer for the insightful comment and for providing two highly relevant references. Both Gil-García et al. (2023)[10] *and Gil-García et al. (2022)*[11] *present sophisticated GIS-based MCDM approaches that model turbine-level wake interactions to support optimal site selection for offshore wind farms. We acknowledge the value of these studies and have now cited them in the revised manuscript.*

It is also worth mentioning that our study addresses a different research goal. While the above studies are focused on identifying the best locations for turbine placement, our objective is to evaluate national-scale offshore wind power generation potential, incorporating wake-induced efficiency losses into the LCOE calculations. Rather than focusing on micro-siting optimization, we assess how wake losses affect broader economic viability under different technical layouts and climate scenarios.

In this context, we note that Pryor et al. [12] *made important contributions by using mesoscale meteorological simulations to quantify the spatial extent and persistence of wake effects from large offshore wind clusters. Building on this understanding, our study takes the next step by embedding wake-induced losses into a techno-economic modeling framework—explicitly linking wake losses with cost structures, turbine technologies, and layout strategies. This allows us to quantify not just power deficits, but also their economic implications through LCOE metrics at the national scale.*

To make this clearer to the reader, we have revised the main text accordingly and included all relevant citations.

What about other designs, for example, staggered wind turbines?

Thank you for your insightful comment regarding other wind turbine designs, such as staggered layouts. While we acknowledge that staggered turbine arrangements could be an important consideration in wake effect analysis, currently our study focuses primarily on regularized turbine configurations, which is the main scope of our research. However, we recognize that staggered designs may offer valuable insights, and we will mention this as a potential area for

future research. Thank you again for raising this point.

Discussion:

The discussion is critical, but what's happening in the rest of the world? Could it serve as a reference for China? Is it possible to adopt strategies from other countries to guide their development in offshore wind energy?

Thank you for your thoughtful question regarding the global context of our research. While the focus of our study is China, we do recognize the value of international perspectives on informing China's strategy. In the discussion, we have added new part on global offshore wind developments, drawing comparisons between China and leading offshore wind countries such as the UK, Germany, and Denmark. We also highlighted those transferable strategies not only limited to China - including floating wind technology advancements, policy frameworks, and spatial planning practices. These additions aim to provide an international perspective and strengthen the broader significance of our findings. Please see the revised section in the Discussion (page 9).

Reviewer #3 (Remarks on code availability):

The materials and methods are sufficient to reproduce the article.

We are glad that our work is reproducible!

Reviewer #4 (Remarks to the Author):

Comments on Main Body

Lines 121–128:

The significantly lower potential obtained in your study appears to be primarily due to the reduced usable area compared to previous studies. I believe your assessment is more realistic than others, as it is impractical to assume that every ocean space can accommodate offshore wind turbines (such as in S1 and S2 from the case studies). However, making a direct comparison between your results and previous literature is challenging without a clear understanding of the assumptions in those studies. I suggest presenting a comparative table that outlines key methodological differences between your study and the literature (8, 9, 11, 17, 23). This table could include details such as usable area, wind speed data sources, turbine spacing assumptions, total number of turbines, turbine rating, capacity density (MW/km²), and wake effect methods (e.g., Gaussian, cumulative curl, Jensen, or assumed wake loss ratio). Providing this structured comparison would offer a clearer perspective on the novelty of your work, rather than focusing solely on differences in TWh/year, which are largely influenced by the reduction in usable area.

Thank you for your constructive suggestion. We agree that directly comparing our results with those of previous studies can be confusing without a clear understanding of the underlying assumptions and methodologies. Your suggestion to include a comparative table is an excellent idea, as it would allow for a structured and transparent comparison of key methodological differences between our study and the literature.

In response, we have included such a table that outlines these critical variables. By presenting these key differences, we aim to further highlight the novelty and realistic nature of our work, particularly in terms of the reduced usable area and more practical assumptions regarding the deployment of offshore wind turbines. Thank you once again for this insightful suggestion, which will significantly improve the quality of our manuscript.

Table 2. Comparative overview of offshore wind potential assessments in China

	Davidson MR [6]	Sherman P [3]	Yang Wang [7]	Xi Deng [8]	Kelly Eurek [9]	This Study
Usable Area (M km2)	-	0.4407*	0.477*	-	0.728*	0.4396-0.6423
Wind Speed Data	MERRA2	MERRA2	NCC-CMA	CCMP	NCAR	MERRA2/CMIP
Turbine Spacing	9D*9D	7D*7D	10D*5D	7D*7D	10D*5D	10D*5D
Total Number of Turbines (k)	-	423.65*	321.47*	-	1040.00*	69.264-294.4
Turbine Rating (MW)	5	8	6/6.7/8	10	3.5	4/8/11
Capacity density (MW/km^2)	5	7.69*	4.726*	9.0909*	5	1.2153-2.6186
Wake effect methods	0	5%	0	-	10%	Gaussian, Cumulative curl, Jensen
Capacity potential (TW)	-	3.39	2.254	-	3.64	0.7619-1.4032

*Note: Values marked with * are computed based on the data provided in the respective papers.*

The table highlights the following points:

- *Usable Area: Our study calculates a range of usable areas, while some previous studies did not provide this data. Our calculations also take into account the differences between floating and bottom-fixed wind farms, providing more realistic estimates of usable area.*
- *Wake Effects: In our study, we explicitly model wake losses using three methods (Gaussian, Cumulative Curl, and Jensen). Many previous studies either ignored wake losses or applied simplified coefficients.*
- *Turbine Spacing: Our study adopted more realistic turbine spacing based on remote*

sensing data and actual wind farm configurations.

- *Capacity Potential: The capacity potential in our study is notably lower, primarily due to the careful inclusion of wake effects and the reduced usable area. Although our potential is lower than some previous studies, we believe it provides more realistic and actionable insights.*

We provide explanations for the less common datasets used:

- *CCMP (ASA's Cross-Calibrated Multi-Platform Wind Vector Data): This dataset is used for estimating offshore wind resources. It is version 2.0 and is based on cross-calibration from multiple platforms, providing global wind speed data.*
- *NCC-CMA (National Climate Center of the China Meteorological Administration): This dataset, developed by the National Climate Center of China Meteorological Administration, has a high resolution of 3 km × 3 km and provides hourly wind data covering the period from 1980 to 2020.*
- *NCAR (National Center for Atmospheric Research): This dataset is used for NREL's global wind resource supply curves. It is based on a 21-year climate reanalysis dataset (1985-2005) with high temporal and spatial resolution*

Additionally, we have updated the relevant sections in both the main body and Supplementary Information (SI) of the manuscript to reflect these changes and provide further clarification on the methodological differences.

Lines 379–386:

Your rationale for selecting the number of rows and spacing between turbines based on prevailing wind directions is well-founded. I understand that the number of columns was determined using wind farm sizes from IRENA. Could you provide a visual example of Chinese offshore wind farms with a similar number of columns as those in the 3×(Large) layout cases for your 3-row and 4-row configurations? In Europe and the U.S., such large numbers of columns are uncommon, but this may be different in China. Including such a reference would help validate the realism of these layouts.

Thank you for the question. In response, we have included two visual examples that clearly demonstrate the turbine layouts for both the 3-row and 4-row configurations in China. Specifically, we show Fig. 1 for the 3-row configuration (Sheyang H2) and Fig. 2 for the 4-row configuration (CTGNE Yangjiang Shapa - phase II). These figures show that Chinese offshore wind farms do have the 3×(Large) and 4×(Large) layout cases, to validate the realism of our layouts.

We have also provided shapefiles (SHP), which can be accessed through a through a Figshare collection (http://dx.doi.org/10.6084/m9.figshare.29892365). The collection is named "Offshore Wind Turbine Layouts - China". In addition, the 4C Offshore platform offers a detailed map of the wind farms used in this study, which can be accessed via the following links for further verification.

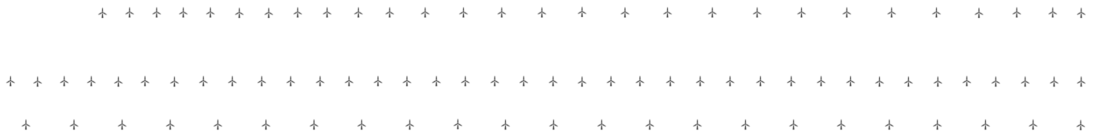

Fig. 1: Sheyang H2 (3-row configuration) – Link to 4C Offshore page

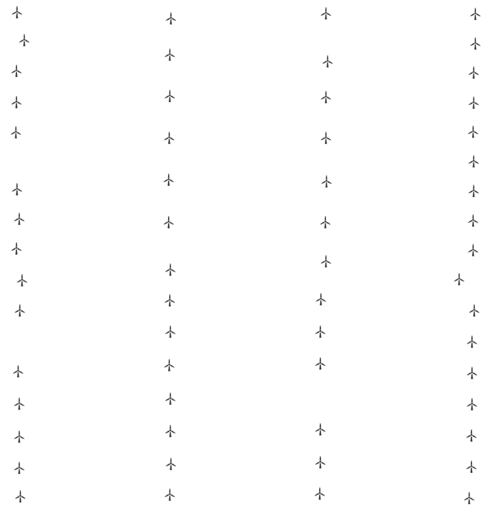

Fig. 2: CTGNE Yangjiang Shapa - phase II (4-row configuration) – Link to 4C Offshore page

We believe these additions will strengthen the manuscript's credibility and provide a clearer understanding of the turbine layouts in China.

Line 428:

Please ensure consistency by using "CapEx" instead of "CapeEx" throughout the "Calculation of Costs" section.

Thank you for pointing out the inconsistency. We have revised the manuscript to ensure consistency by using "CapEx" throughout the "Calculation of Costs" section.

Lines 442–447:

• Please define "n" explicitly (I assume it refers to the project design life) and specify the value

used.

Thank you for your comment. We have revised the manuscript to explicitly define "n" as the project design life, which is assumed to be 25 years for the wind farm's operational lifetime.

• Line 446: The provided formula corresponds to the capital recovery factor, not the Fixed Charge Rate (FCR). The FCR is the capital recovery factor multiplied by the project finance factor. In the latest Cost of Wind Energy Review, we assumed a project finance factor of 105% (see NREL Report). Please incorporate this factor into your calculation and state your assumption.

We thank the reviewer for this important correction. You are absolutely correct that our equation (9) represents the capital recovery factor rather than the complete Fixed Charge Rate. We have revised our methodology to incorporate the project finance factor following NREL's approach.

The corrected FCR calculation is now:

$$FCR = CRF \times PFF$$

Where:

CRF = *Capital Recovery Factor* = $WACC / [1 - (1 + WACC)^{-n}]$

PFF = *Project Finance Factor* = 1.05 *(105%, following NREL's 2022 Cost of Wind Energy Review)*

We have updated the equations in the manuscript and recalculated all LCOE values accordingly. This correction ensures the methodological consistency with established wind energy economic assessment practices.

• Line 447: Is the Weighted Average Cost of Capital (WACC) a nominal or real value? This distinction is important, as it determines whether the FCR should be real or nominal. At NREL, we typically use the real FCR for LCOE calculations.

Thank you for your comment regarding the distinction between nominal and real values. Based on the formulas presented in the manuscript:

- *Equation (1) calculates the nominal WACC, which takes into account the cost of debt adjusted for the corporate tax rate and the cost of equity, weighted by the equity and debt ratios.*

$$norm\_WACC = \text{Equity and Debt ratio} * \text{Cost of debt} * (1 - \text{coporate tax rate}) + (1 - \text{Equity and Debt ratio}) * \text{Cost of equity} \quad (1)$$

The values used in the WACC and FCR calculations are based on the financial model established by Luo et al.[13]*, which provides a detailed analysis of wind and solar energy projects in China. Specifically:*

Cost of debt and equity ratio: The 80:20 debt-to-equity ratio and cost of debt (5-6%) were derived from real financial documents for wind projects in China.

Cost of equity: The 8% cost of equity was based on financial data from five wind projects.

- *Equation (2) then adjusts the nominal WACC to derive the real WACC. This is done using the inflation rate, as indicated in the formula:*

$$Real\_WACC = (1+norm\_WACC)/(1+inflation)^{-1} \qquad (2)$$

This conversion from nominal to real WACC is critical for accurate financial calculations when adjusting for inflation.

As per your suggestion, we have used the real FCR in our LCOE calculations, in alignment with the typical practices at NREL. This adjustment ensures that the calculations remain consistent with inflation adjustments and provide a more realistic representation of future costs. Using the assumed 80 % debt / 20 % equity structure, 5.5 % cost of debt, 8 % cost of equity, a 25 % corporate tax rate and a 2.0% inflation rate, the nominal WACC is 4.9 % and the real WACC is 2.84 %. For a 25-year project life, this yields a real fixed-charge rate of 5.86 %. See the table below for details.

Table 3. Fixed-bottom and floating offshore wind project financial assumptions

Parameter	Nominal Value	Real Value
Weighted average cost of capital	4.9%	2.84%
Capital recovery factor	6.63%	5.58%
Fixed charge rate	6.96%	5.86%

By above, we clearly distinguish this point in our paper to ensure that the definitions used for WACC and FCR are clear and consistent with industry standards.

Line 451:

• Are array cable costs included in the analysis? If so, please clarify, as these costs vary based on layout choice.

Thank you for your valuable comments. In response to your question regarding the inclusion of array cable costs in the analysis, we have indeed considered the costs associated with array

cables. We referred to the layout from the Orbit document, specifically the Grid layout with no partial strings type (Fig.3) and used typical layouts as shown in the schematic diagrams in Fig. 4 and Fig. 5.

[REDACTED]

Fig. 3. Grid layout with no partial strings

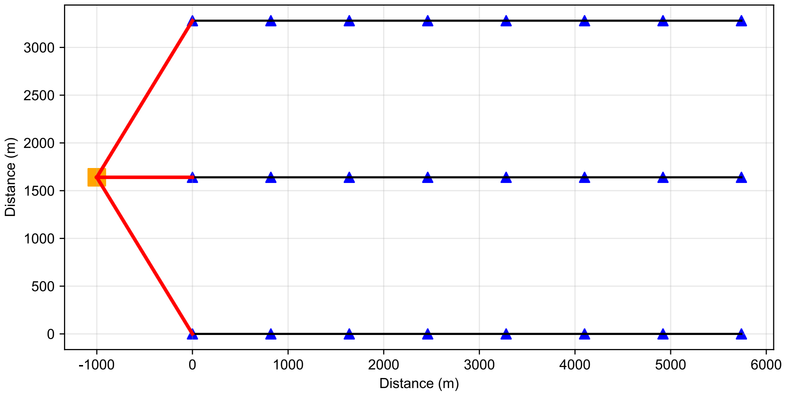

Fig. 4. 3-row, 20-column Layout Cable Distribution

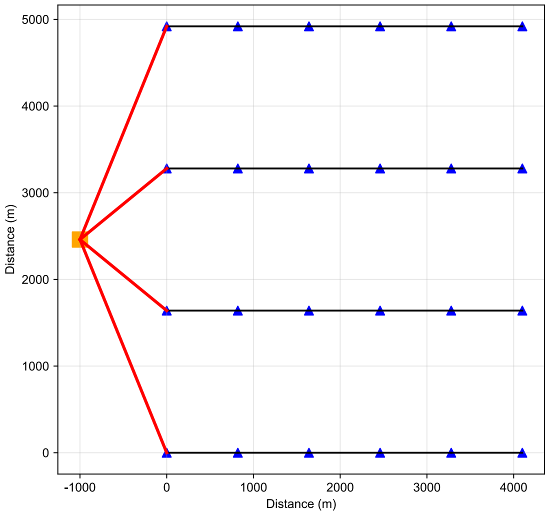

Fig. 5. 4-row, 15-column Layout Cable Distribution

The information regarding the array cables for each layout type is also listed as follows:

Table 4. Array cable lengths by layout type and turbine model

**Layout Type**	**Turbine Model**	**Internal Cables (km)**	**Main Cables (km)**	**Total Cables (km)**
3-row Small, 8 columns	SWT_4_130	13.65	4.28	17.93
3-row Small, 20 columns	SWT_4_130	37.05	4.28	41.33
3-row Small, 33 columns	SWT_4_130	62.4	4.28	66.68
4-row Small, 6 columns	SWT_4_130	13	6.77	19.77
4-row Small, 15 columns	SWT_4_130	36.4	6.77	43.17
4-row Small, 25 columns	SWT_4_130	62.4	6.77	69.17
3-row Medium, 8 columns	V164_8	17.22	4.84	22.06
3-row Medium, 20 columns	V164_8	46.74	4.84	51.58
3-row Medium, 33 columns	V164_8	78.72	4.84	83.56
4-row Medium, 6 columns	V164_8	16.4	7.9	24.3
4-row Medium, 15 columns	V164_8	45.92	7.9	53.82
4-row Medium, 25 columns	V164_8	78.72	7.9	86.62

**Layout Type**	**Turbine Model**	**Internal Cables (km)**	**Main Cables (km)**	**Total Cables (km)**
3-row Large, 8 columns	EW_11_208	21.84	5.62	27.46
3-row Large, 20 columns	EW_11_208	59.28	5.62	64.9
3-row Large, 33 columns	EW_11_208	99.84	5.62	105.46
4-row Large, 6 columns	EW_11_208	20.8	9.44	30.24
4-row Large, 15 columns	EW_11_208	58.24	9.44	67.68
4-row Large, 25 columns	EW_11_208	99.84	9.44	109.28

We hope this answers your query and appreciate your detailed review and feedback.

• Does C_found include the transition piece for monopiles? Please specify.

Thank you for your insightful question. In our current manuscript, the transition piece is not explicitly accounted for in the cost breakdown of the monopile substructure. However, based on industry practices and information from the referenced literature (such as Myhr et al.[14], Bjerkseter[15],), the transition piece is typically included in the overall mass and cost of the monopile structure. For instance, in Myhr et al[14]., the monopile substructure is calculated with a total mass of 1200 tons, which includes the monopile itself and the transition piece.

We have updated the manuscript to clarify that the transition piece is inherently included in the monopile cost, consistent with standard practices. We have revised the manuscript accordingly to provide more transparency on this matter.

• Does C_ins cover the installation costs for components beyond the foundation (e.g., turbines, substations, array cables, export cables)? Please clarify.

We thank the reviewer for raising this important point. We confirm that C_ins in our model does include the installation costs for components beyond the foundation, such as:

- *Wind turbines: The costs are calculated based on the number of turbines and the installation process, which includes turbine assembly, tower installation, and connection to the electrical system.*
- *Substations: These are included based on the assumption of the offshore wind farm needing one or more substations to convert the generated power to a grid-compatible voltage.*
- *Array cables: The costs for both inter-array cables (for wind farm internal connections) and export cables (connecting the farm to the onshore grid) are also accounted for in the C_ins estimation.*

The cost estimation methodology for these installation components follows established approaches in the offshore wind literature. The comprehensive cost framework for turbines and electrical infrastructure draws from standardized offshore wind cost assessment methodologies (Bjerkseter & Ågotnes[15], Bosch et al.[4]). For floating offshore wind configurations, we additionally incorporate specialized installation considerations following Maienza et al.[16]. The formulas for calculating installation costs in our model are based on typical offshore wind industry standards for turbine installation and electrical infrastructure, and we update these for each scenario (e.g., based on port distances and transmission distance).

We have clarified these details in the revised manuscript, and the corresponding formulas for turbine and infrastructure installation are now explicitly included in the Methods section.

• It appears that soft CapEx elements such as construction financing, installation contingency, procurement contingency, and construction insurance are not considered. Please either include these costs or explicitly state that they are excluded from the LCOE calculations. If needed, NREL's open-source model contains relevant formulas and industry-based factors (see ORBIT Code).

We appreciate the reviewer's valuable comment regarding the absence of soft CapEx elements in our LCOE calculations. We have now incorporated these costs into our model as suggested.

We have implemented a comprehensive soft cost calculation methodology based on NREL's ORBIT (Offshore Renewable Balance-of-system Installation Tool) framework, following the latest factors from Stehly et al.[2]. The following soft CapEx elements have been integrated:

- *Construction Financing: We have implemented a time-dependent cost of capital calculation based on actual expenditure schedules from U.S. projects. The model uses an interest during construction rate of 0.044 and a tax rate of 0.26, applied to a yearly spending profile (Year 0-25%, Year 1-25%, Year 2-30%, Year 3-10%, Year 4-10%, Year 5-0%) to calculate the financing factor.*

- *Installation Contingency: Following ORBIT/NREL methodology, we have applied a factor of 3.45% (0.0345) to the total installation costs.*
- *Procurement Contingency: We have implemented a 5.75% (0.0575) factor applied to non-installation capital expenditures (turbine, BOS, and project costs).*
- *Construction Insurance: A factor of 1.15% (0.0115) has been applied to calculate all-risk property, delays in start-up, third-party liability, and broker's fees.*

Additionally, we have included other relevant soft costs, such as commissioning costs (1.15%) and decommissioning costs (17.5% of installation costs).

Through case analysis, the impact of soft costs on LCOE is significant. In our test case, the LCOE calculation including soft costs is approximately 10-15% higher than the result without soft costs, indicating that omitting soft costs leads to underestimation of LCOE.

Line 665:
Your LCOE values for floating and fixed-bottom technologies fall within a reasonable range, considering this is a resource assessment. However, please refer to my earlier comments on refining your assumptions in both the main body and supplemental information.

Thank you for your valuable comments. In response, we have made a series of clarifications and updates to both the main text and the Supplementary Information to ensure methodological transparency and reproducibility. These refinements include:

- *A clear specification of the components included in both the foundation cost term (C_found) and installation cost term (C_ins), disaggregating costs associated with turbines, substations, array cables, and export cables;*
- *The explicit inclusion of previously omitted soft CapEx elements, such as financing costs, installation and procurement contingencies, and construction insurance. These have been incorporated using standard proportional multipliers based on the NREL ORBIT framework, and are now documented in the updated methods;*
- *Clarification that cable costs are layout-specific, with both array and export cable lengths derived from modeled farm geometries and accounted for in the total CapEx;*
- *A clearer breakdown of installation scopes, distinguishing between turbine, substructure, and electrical infrastructure installation activities.*

We believe that these updates significantly enhance the robustness and transparency of our economic modeling framework. Once again, we thank the reviewer for their constructive feedback, which has helped improve the quality and credibility of our LCOE assessment.

Line 676:

Did all literature sources (8, 9, 11, 17, 23) assume a 10% wake loss ratio? If different papers used varying wake loss ratios, I recommend incorporating those values into your plots. Highlighting differences in wake loss assumptions may further emphasize the novelty of your work.

Thank you for your suggestion regarding the wake loss ratio. You are correct that the referenced studies (8, 9, 11, 17, 23) used varying wake loss assumptions—some studies assumed 0% wake losses, others used 5% or 10%, and several did not consider wake effects at all in their potential assessments (see Table 2). In our study, we deliberately chose to use the commonly cited 10% wake loss ratio as a reference point to highlight how previous studies may have underestimated the impact of wake effects on offshore wind potential. To better illustrate this point and emphasize the novelty of our approach, we have revised the manuscript to clearly specify the wake loss assumptions used in each reference and incorporate comparison plots showing potential estimates under 0%, 5%, and 10% wake loss scenarios. This revision will demonstrate how our more realistic wake loss calculations (which we found to be considerably higher than 10%) compare to these conventional assumptions, providing readers with a clearer understanding of how different wake loss assumptions significantly influence offshore wind potential estimates and further highlighting the importance of using more sophisticated wake modeling approaches.

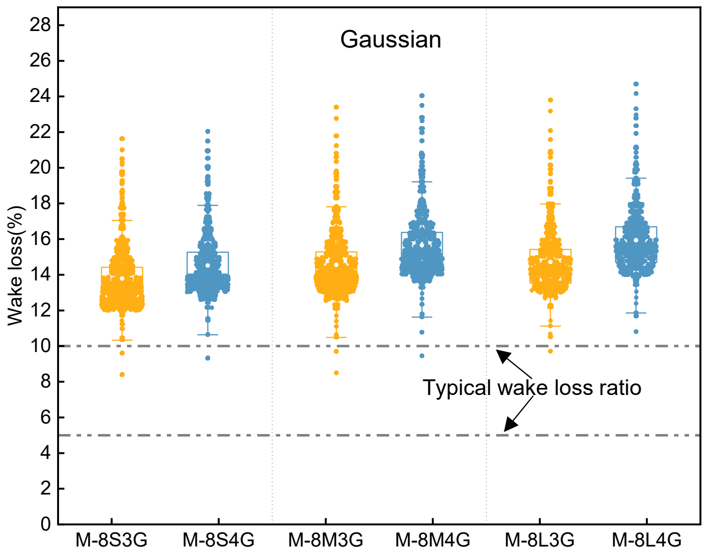

Fig. 6. Revised wake loss comparison diagram

Comments on Supplemental Information

Line 54:

How are the distances (D) calculated? Is this based on the closest point to shore? Do you have a predefined set of potential Points of Interconnection (POI), and is transmission distance determined using the nearest POI? Similarly, for ports, do you calculate distance to the closest port? If D represents both transmission distance and distance to port and is based solely on the nearest shoreline, please acknowledge this simplification. Additionally, clarify that installation costs are influenced by port distance, while transmission costs depend only on transmission distance.

Thank you for your insightful question regarding the calculation of distances (D). In our study, the distance is calculated based on the nearest distance from each wind turbine to the shore, without considering predefined potential Points of Interconnection (POI). We acknowledge this simplification and have clarified this in the manuscript. Additionally, we have also noted that installation costs are influenced by the distance to the nearest port, while transmission costs are determined solely by the transmission distance, which is based on the closest point to shore.

We have added explanations in the Supplementary Methods section saying that: "D represents distance from shore, both unit is m. Here, D is calculated as the shortest distance from each turbine location to the nearest shoreline and is used as a proxy for both port distance (for installation cost estimation) and transmission distance (for grid connection cost estimation). We acknowledge that this simplification may not fully represent real-world infrastructure layouts involving predefined ports or Points of Interconnection (POIs), but it provides a practical approximation for large-scale analysis."

Line 69:

The CapEx estimates derived from this formula fall within an acceptable range, at least for the U.S. offshore wind market. Could you provide a reference or background information justifying the use of this formula?

We thank the reviewer for the comment. The capital expenditure (CapEx) formula used in our analysis is adapted from the work of Maienza et al.[16], specifically Section 2.1.1 of their study on floating wind farm component costs. In their framework, the wind turbine cost is modeled as a linear function of rated capacity, derived from regression analysis of real project data. This relationship is defined as:

$$C_{turb} = (1.6 \cdot p_T - 1.9) \cdot \frac{1.1789}{p_T} \tag{3}$$

where p_T is the rated power in MW and 1.1789 is a scaling coefficient we apply to convert their original cost values to 2022 USD, accounting for inflation and exchange rate adjustments.

We have now clarified this reference and rationale in the revised Methods section.

Line 81:

Does this cost estimate include the transition piece (TP), or are you assuming a TP-less monopile? The values seem reasonable, but could you provide a reference or the rationale behind this formula?

We appreciate this comment. The cost estimate for monopile foundations in our analysis includes the transition piece (TP), as the TP is considered an integral part of the monopile structure for offshore wind farms. This assumption is consistent with industry standards and literature, such as Myhr et al.[14] *and Bjerkseter*[15]*. In the revised manuscript, we have explicitly stated that the TP is included in the monopile cost estimate and provided appropriate references.*

Line 83:

Similar to the monopile comment—please provide a reference for this cost estimate.

The cost estimate for jacket foundations, similar to monopiles, is derived from cost models used in the industry, including references from Myhr et al.[14] *and Bjerkseter*[15]*. These sources provide cost breakdowns based on water depth, material costs, and installation complexity. We have updated the manuscript to explicitly reference these sources and clarify the assumptions behind our jacket cost estimate.*

Line 85:

Floating substructure costs are nearly as high as turbine CapEx. While the estimated costs appear somewhat low, they remain within a reasonable range. Could you provide a reference or background explanation for this formula?

Thank you for the feedback. Our floating substructure cost estimates may appear somewhat low compared to current industry costs, our values are based on projections from Maienza et al[17] *and Myhr et al.*[14]*, which report similar costs for floating platform concepts like WindFloat and Tension-Leg Platforms (TLPs). Then we adjusted downward to account for expected cost reductions due to technological improvements and economies of scale as the floating wind industry matures. This approach reflects the historical cost decline patterns observed in other offshore wind technologies. We have revised the manuscript to better explain this methodology and provide additional references supporting our cost projection assumptions.*

Lines 95–96:

Both cost estimates seem largely insensitive to transmission distance, which is unexpected. For

example, AC transmission costs at 10 km and 1000 km are nearly identical ($34.5/kW vs. $34.51/kW). Could there be an issue with the formula? Please verify.

Thank you for pointing this out. Upon further review, we have identified an error in our formula. The issue arose from the incorrect handling of the transmission distance D, which caused both HVAC and HVDC transmission costs to be largely insensitive to distance, leading to unrealistic results.

We have corrected the formulas to ensure proper handling of the transmission distance. The corrected formulas for the two transmission methods are as follows:

HVAC (D ≤ 56000):

$$C_{inst}(D) = (7.598D + 50814.98) \tag{4}$$

HVDC (D > 56000):

$$C_{inst}(D) = (1.966D + 346764.58) \tag{5}$$

where D is the transmission distance in meters.

Additionally, we conducted a sensitivity test to validate the corrected formulas, the updated test results are as Table 5.

Table 5. Transmission Cost Sensitivity Analysis Results (Corrected Formulas)

Distance form shore (km)	Value($/kw)
10	126.80
50	430.73
60	476.47
100	543.43

As shown in the table above, the corrected formulas now realistically reflect the transmission costs as they scale with distance.

Further in the revised cost modeling, we further incorporated a distinction between internal array cables and export cables. The two components are modeled separately: internal cable lengths are determined by typical farm layouts (as detailed in our earlier response on array cable configuration), while export cable costs are based on the offshore transmission distance D and layout configuration. Full details of this implementation are provided in the updated "Transmission Costs" section of the Supplementary Information (SI).

We greatly appreciate your careful review and valuable feedback, which helped us correct this mistake.

Lines 106–111:

If these values represent only the substructure installation costs, they appear reasonable. However, if they account for the entire wind farm’s installation, they seem considerably low. The 2024 Cost of Wind Energy Review estimates installation costs at $830/kW for fixed-bottom and $1,058/kW for floating, assuming port distances of 116 km and 189 km, respectively. Please provide a reference or justification for these formulas.

We appreciate the reviewer's observation. The values provided in our study represent only the substructure installation costs, not the entire wind farm installation. We have now clarified this distinction in the manuscript. Our substructure installation cost formulas are based on established methodologies from Maienza et al[17], Myhr et al.[14] and Bjerkseter & Ågotnes[15]. For comparison, our study estimates installation costs at $358 USD/kW for fixed-bottom wind farms (similar to NREL configurations), which shows acceptable agreement with the $284 USD/kW value presented in the appendix. It should be noted that our current model does not account for port distance effects on installation costs, which is a factor considered in the NREL study (116 km for fixed-bottom and 189 km for floating). We plan to address this limitation in following research to provide more comprehensive cost modeling.

Lines 112–117:

What is the basis for using 2.3%? In NREL’s open-source model (ORBIT), this value accounts for site auction prices, site assessment plans, construction plans, and installation plans. Our assumptions can be found here: ORBIT Code. Please provide a reference or rationale for your assumption.

Thank you for your valuable suggestions. We based our use of the 2.3% figure on the "Development and Project Management" cost ratio from NREL's 2021 Cost of Wind Energy Review report for fixed-bottom offshore wind. This value was selected because it is the highest among the figures reported in both the 2021 and 2022 NREL reports. Upon reviewing other literature, we found that this cost category was often higher in some studies, so we opted for the maximum value to ensure a more conservative estimate.

Additionally, in response to the reviewer's feedback, we cross-referenced NREL's open-source ORBIT model, using its default values to calculate the $/kW costs for wind farms with capacities of 800 MW and 600 MW. The results were $231/kW and $309/kW, respectively. These values differ significantly from the 2024 report value of $123/kW and the 2022 report value of $91/kW.

We further consulted works by A. Martinez et al[17], Kangxin An[18], and others, who define the development and consent (D&C) phase costs as including environmental surveys, met station and seabed station assessments, as well as project management and development services. Their estimated D&C costs for bottom-fixed offshore wind came to $210/kW.

After comparing these data sources, we decided to adopt the value from A. Martinez et al[17]. and adjusted it to $225/kW for 2022 by applying a wind cost depreciation rate and inflation adjustment. This aligns with the convention in NREL reports, where both fixed and floating offshore wind share the same development costs.

We have updated the relevant sections of the paper accordingly. Once again, thank you for your insightful feedback, which has helped to strengthen our analysis.

Lines 118–124:
Similarly, what is the basis for using 2.4%? In ORBIT, we assume 17.5% of installation CapEx (see ORBIT Code). Please provide a reference or justification.

We appreciate the reviewer's inquiry. This value was derived by referencing typical case proportions in NREL's 2022 Cost of Wind Energy Review. We previously validated that our overall value was similar to the 17.5% of installation CapEx approach used in ORBIT[19].

However, as mentioned in our previous response to your comment on soft costs, we are revising this aspect of our model. We will modify this component to properly place it within the soft cost framework and will explicitly use the 17.5% of installation CapEx ratio as implemented in the ORBIT code. The standardization to ORBIT's approach will enhance the comparability of our results with other studies in the field.

Lines 126–136:
The cost appears to be largely insensitive to D. Could you provide a reference or explain the reasoning behind these formulas?

Thank you for this insightful question regarding the apparent low sensitivity of O&M costs to distance D in our formulas. Our O&M cost model follows the methodology established by Bosch et al.[4], as cited in line 127, where the formulas are derived from their comprehensive analysis of offshore wind O&M costs across varying distances and foundation technologies. The relatively low sensitivity to distance D reflects the actual cost structure of offshore wind O&M operations, where the large constant terms (37,292 for monopile/jacket and 39,191 for floating) represent substantial base O&M costs that are independent of distance, including scheduled maintenance activities, component replacement costs, insurance, monitoring systems, and operational overhead. The smaller linear coefficients (0.0212 and 0.0433) capture the incremental costs primarily associated with extended vessel transit times, increased fuel consumption, and additional weather window constraints for distant sites.

As illustrated in our Fig.7, while there is a clear upward trend with distance, the effect is modest

compared to the technology-dependent base costs. Most O&M strategies rely on planned maintenance campaigns rather than frequent trips, which reduces the relative impact of distance on total costs. We appreciate your attention to this important aspect of our model and acknowledge that more detailed O&M cost modeling could provide additional insights, particularly regarding the optimization of maintenance strategies for different distance ranges. This represents an important area for future research, and we plan to investigate more granular O&M cost dependencies in subsequent studies.

[REDACTED]

Fig. 7. Offshore wind O&M cost sensitivity to distance from coast

Reviewer #5 (Remarks to the Author):

I co-reviewed this manuscript with one of the reviewers who provided the listed reports. This is part of the Nature Communications initiative to facilitate training in peer review and to provide appropriate recognition for Early Career Researchers who co-review manuscripts.

Thank you for co-reviewing our paper, we hope our revisions satisfy you.

References

1. IRENA. *Renewable Power Generation Costs in 2022*. (2023).
2. Stehly, T., Duffy, P. & Hernando, D. M. 2022 Cost of Wind Energy Review. (2023) doi:10.2172/2278805.
3. Sherman, P., Chen, X. & McElroy, M. Offshore wind: An opportunity for cost-competitive decarbonization of China's energy economy. *Science Advances* **6**, eaax9571 (2020).
4. Bosch, J., Staffell, I. & Hawkes, A. D. Global levelised cost of electricity from offshore wind. *Energy* **189**, 116357 (2019).
5. Xu, Z., Han, Y., Tam, C.-Y., Yang, Z.-L. & Fu, C. Bias-corrected CMIP6 global dataset for dynamical downscaling of the historical and future climate (1979–2100). *Sci Data* **8**, 293 (2021).
6. Davidson, M. R., Zhang, D., Xiong, W., Zhang, X. & Karplus, V. J. Modelling the potential for wind energy integration on China's coal-heavy electricity grid. *Nat Energy* **1**, 1–7 (2016).
7. Wang, Y., Chao, Q., Zhao, L. & Chang, R. Assessment of wind and photovoltaic power potential in China. *Carb Neutrality* **1**, 15 (2022).
8. Deng, X. *et al.* Offshore wind power in China: A potential solution to electricity transformation and carbon neutrality. *Fundamental Research* S266732582200440X (2022) doi:10.1016/j.fmre.2022.11.008.
9. Eurek, K. *et al.* An improved global wind resource estimate for integrated assessment models. *Energy Economics* **64**, 552–567 (2017).
10. Gil-García, I. C., Ramos-Escudero, A., Molina-García, Á. & Fernández-Guillamón, A. GIS-based MCDM dual optimization approach for territorial-scale offshore wind power plants. *Journal of Cleaner Production* **428**, 139484 (2023).
11. Gil-García, I. C., Ramos-Escudero, A., García-Cascales, M. S., Dagher, H. & Molina-García, A. Fuzzy GIS-based MCDM solution for the optimal offshore wind site selection: The Gulf of Maine case. *Renewable Energy* **183**, 130–147 (2022).
12. Pryor, S. C., Barthelmie, R. J. & Shepherd, T. J. Wind power production from very large offshore wind farms. *Joule* **5**, 2663–2686 (2021).
13. Lou, J., Yu, S., Cui, R. Y., Miller, A. & Hultman, N. A provincial analysis on wind and solar investment needs towards China's carbon neutrality. *Applied Energy* **378**, 124841 (2025).
14. Myhr, A., Bjerkseter, C., Ågotnes, A. & Nygaard, T. A. Levelised cost of energy for offshore floating wind turbines in a life cycle perspective. *Renewable Energy* **66**, 714–728 (2014).
15. Bjerkseter, C. & Ågotnes, A. (2013) - Levelised Costs of Energy for Offshore Floating

Wind Turbine Concepts.
16. Maienza, C. *et al.* A life cycle cost model for floating offshore wind farms. *Applied Energy* **266**, 114716 (2020).
17. Martinez, A. & Iglesias, G. Multi-parameter analysis and mapping of the levelised cost of energy from floating offshore wind in the Mediterranean Sea. *Energy Conversion and Management* **243**, 114416 (2021).
18. An, K., Cai, W., Lu, X. & Wang, C. High-resolution gridded dataset of China's offshore wind potential and costs under technical change. *Sci Data* **12**, 69 (2025).
19. Nunemaker, J., Shields, M., Hammond, R. & Duffy, P. *ORBIT: Offshore Renewables Balance-of-System and Installation Tool*. NREL/TP-5000-77081, 1660132, MainId:26027 https://www.osti.gov/servlets/purl/1660132/ (2020) doi:10.2172/1660132.
